# A murine neonatal model of necrotizing enterocolitis caused by anemia and red blood cell transfusions

**DOI:** 10.1038/s41467-019-11199-5

**Published:** 2019-08-02

**Authors:** Krishnan MohanKumar, Kopperuncholan Namachivayam, Tanjing Song, Byeong Jake Cha, Andrea Slate, Jeanne E. Hendrickson, Hua Pan, Samuel A. Wickline, Joo-Yeun Oh, Rakesh P. Patel, Ling He, Benjamin A. Torres, Akhil Maheshwari

**Affiliations:** 1 Department of Pediatrics, Morsani College of Medicine, University of South Florida, Tampa, FL 33612 USA; 2 Department of Pediatrics, Johns Hopkins University, Baltimore, MD 21287 USA; 3 Department of Molecular Pharmacology and Physiology, Morsani College of Medicine, University of South Florida, Tampa, FL 33612 USA; 4 Department of Comparative Medicine, University of South Florida, Tampa, FL 33612 USA; 5 Center for Comparative Medicine, Massachusetts General Hospital, Boston, MA 02114 USA; 6 Department of Laboratory Medicine, Yale School of Medicine, New Haven, CT 06520 USA; 7 Department of Pediatrics, Yale School of Medicine, New Haven, CT 06520 USA; 8 Department of Cardiology, Morsani College of Medicine, University of South Florida, Tampa, FL 33629 USA; 9 Center for Free Radical Biology, University of Alabama at Birmingham, Birmingham, AL 35294 USA; 10 Department of Pathology, University of Alabama at Birmingham, Birmingham, AL 35294 USA

**Keywords:** Innate immunity, Paediatric research

## Abstract

Necrotizing enterocolitis (NEC) is an idiopathic, inflammatory bowel necrosis of premature infants. Clinical studies have linked NEC with antecedent red blood cell (RBC) transfusions, but the underlying mechanisms are unclear. Here we report a neonatal murine model to investigate this association. C57BL/6 mouse pups rendered anemic by timed phlebotomy and then given RBC transfusions develop NEC-like intestinal injury with prominent necrosis, inflammation, and submucosal edema/separation of the lamina propria in the ileocecal region and colon within 12–24 h. The anemic intestine is infiltrated by inflammatory macrophages, which are activated in situ by RBC transfusions via a Toll-like receptor (TLR)-4-mediated mechanism and cause bowel injury. Chelation of RBC degradation products with haptoglobin, absence of TLR4, macrophage depletion, and inhibition of macrophage activation is protective. Intestinal injury worsens with increasing severity and the duration of anemia prior to transfusion, indicating a need for the re-evaluation of current transfusion guidelines for premature infants.

## Abstract (web summary)

The development of neonatal necrotising enterocolitis has been temporally associated with red blood cell transfusions in retrospective human studies. Here, the authors develop a neonatal mouse model of necrotising enterocolitis in anaemic mice receiving red blood cell transfusion that recapitulates features of the human condition.

## Introduction

Necrotizing enterocolitis (NEC) is an inflammatory bowel necrosis of premature infants, and is a leading cause of mortality in infants born between 22 and 28 weeks gestation^1^. In the past decade, more than 20 retrospective clinical studies^2–24^ and the meta-analysis of observational data presented in these studies^4,25^ have shown an association between red blood cell (RBC) transfusions and NEC. These studies are constrained by limitations of sample size and center-based differences in demographics, severity of illness, and the baseline rates of NEC, but there are several common elements: (a) 25–40% of all cases developed NEC within 48 h following a RBC transfusion; (b) infants with transfusion-associated NEC were born at an earlier gestational age and with a lower birth weight than those who developed NEC unrelated to transfusions; and (c) many cases had received multiple RBC transfusions^3^. Despite the consistency of this association, the retrospective, observational design of these studies has left unaddressed concerns about causality and the overall quality of evidence^26^. Consequently, there has been a long-standing need for a preclinical model to investigate this association.

In this study, we address these knowledge gaps by developing a neonatal murine model of transfusion-associated NEC. We hypothesized that RBC transfusions may trigger NEC in the presence of severe anemia; the emphasis on anemia was based on several emerging lines of evidence: (a) NEC has been reported in patients with anemia related to diverse neonatal conditions such as glucose-6-phosphate dehydrogenase deficiency, hemolytic disease of the newborn, and twin-to-twin transfusion syndrome;^27,28^ (b) transfusion-associated NEC is usually a late event seen beyond 4 weeks of postnatal age, when these infants are usually anemic;^11^ and (c) intestinal injury has been observed in other populations of critically ill infants such as those undergoing treatment with cardiopulmonary bypass or extracorporeal membrane oxygenation, particularly when they receive top-up transfusions to treat severe anemia^29^. The effect of anemia in risk stratification for transfusion-associated NEC has been described in at least two clinical studies^10,19^. In the following sections, we show that severe anemia in the murine neonate leads to the development of a low-grade inflammatory state in the intestine with prominent macrophage infiltration. Subsequent RBC transfusions activate these macrophages and causes NEC-like intestinal injury.

## Results

### Neonatal murine model of RBC transfusion-associated NEC

**Fig. 1:**
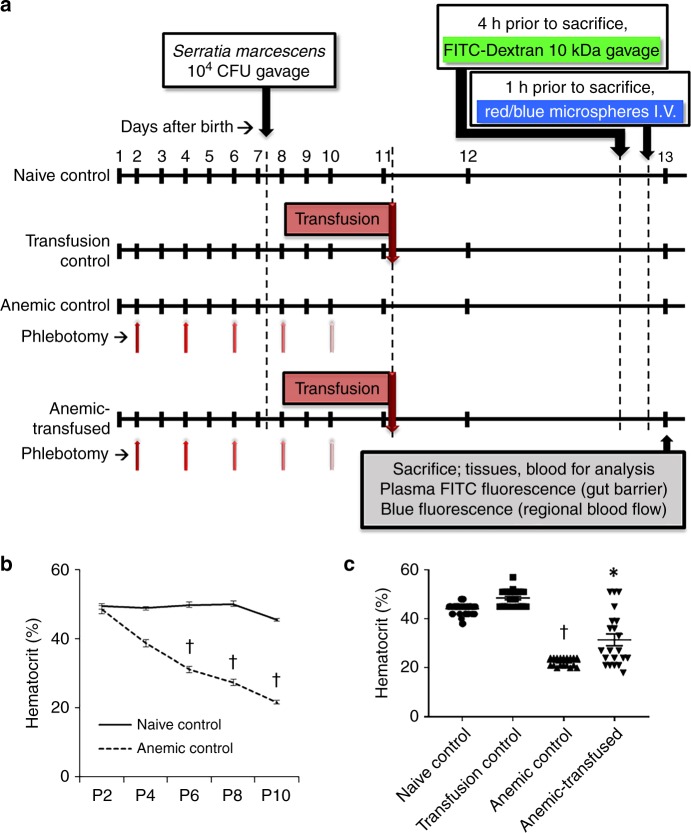
Neonatal murine model of red blood cell (RBC) transfusion-associated necrotizing enterocolitis (NEC)-like injury. **a** Schematic shows the sequence of interventions in naive control, transfusion control, anemic control, and anemic-transfused mice. **b** Longitudinal change in hematocrit in mice subjected to repeated phlebotomy. Line diagrams (means ± standard error, SE) show serial hematocrits in control vs. anemic mice. **c** Hematocrit in the four study groups. Scatter column plots (means ± SE) show the hematocrit values on P12 (24 h after transfusions). Data in **b**, **c** represent *N* = 42 animals in naive control, 21 in transfusion control, 18 in anemic control, and 21 in anemic-transfused groups. Kruskal–Wallis *H* test with Dunn’s post test, *P < 0.05, ^†^*P* < 0.001 vs. naive control

To investigate whether RBC transfusions were detrimental to the neonatal intestine either per se or only when administered in a background of severe anemia, we transfused control and anemic C57BL/6J mouse pups. Thus, the study design included four groups: (a) naive control; (b) transfusion control; (c) anemic control; and (d) anemic-transfused mice (Fig. 1a). In preliminary studies, our mice showed few Gammaproteobacteria in their intestines. Considering the potential importance of enteric Gammaproteobacteria in NEC pathogenesis^30^, we introduced a well-characterized strain of *Serratia marcescens* [10^4^ colony-forming units (CFU) by gavage] in our mice on postnatal day (P) 7 to achieve fecal Gammaproteobacteria abundance similar to premature infants (Supplementary Fig. 1a)^31^. These *Serratia* have been previously used as prototypical Gram-negative bacteria in rodent models of NEC^32,33^ for several reasons: a translational relevance; originally isolated from a premature infant with NEC; (b) non-pathogenic in mice; mice colonized with these bacteria in our laboratory have remained asymptomatic for several months with normal growth and no histological evidence of intestinal inflammation; and (c) natural red pigmentation of *Serratia* colonies facilitates detection in fecal/tissue cultures.

Naive controls were observed over the study period without intervention. The anemic control and anemic-transfused groups were rendered anemic by timed, measured phlebotomy on P2, P4, P6, P8, and P10. Hematocrits and RBC parameters were checked prior to each phlebotomy, and then every 24 h on P11, P12, and P13. In phlebotomized mice, the hematocrit (mean ± standard error, SE) dropped from 49.2 ± 1% on P2 to 21.7 ± 0.4% after phlebotomy on P10 (Kruskal–Wallis *H* test, *p* < 0.001; Fig. 1b). In the transfusion control and anemic-transfused groups, we administered an allogeneic RBC transfusion (20 mL kg^−1^) on P11 from FVB/NJ donors^34^. To recapitulate current clinical practice in premature infants^35^, these RBCs were leukoreduced (buffy coat removal^36^ to eliminate >99% leukocytes and platelets; Supplementary fig. 1b) and packed to a hematocrit of 70%, and to simulate the average storage age of transfused RBCs in these patients, stored at 4 °C for 7 days.

The hematocrits in the naive control, transfusion control, anemic control, and the anemic-transfused groups at the time of sacrifice are depicted in Fig. 1c, and the RBC indices are summarized in Supplementary Table 1. Compared to naive control, the anemic controls showed lower mean corpuscular hemoglobin, mean corpuscular hemoglobin concentration, and reticulocyte hemoglobin. In 8/21 (38.1%) anemic-transfused mice, the hematocrit remained <30% after the first transfusion, and therefore, these pups were transfused again. Animals were sacrificed on P13 or earlier if noted to be in physical distress.

### RBC transfusions trigger NEC-like injury in anemic pups

**Fig. 2:**
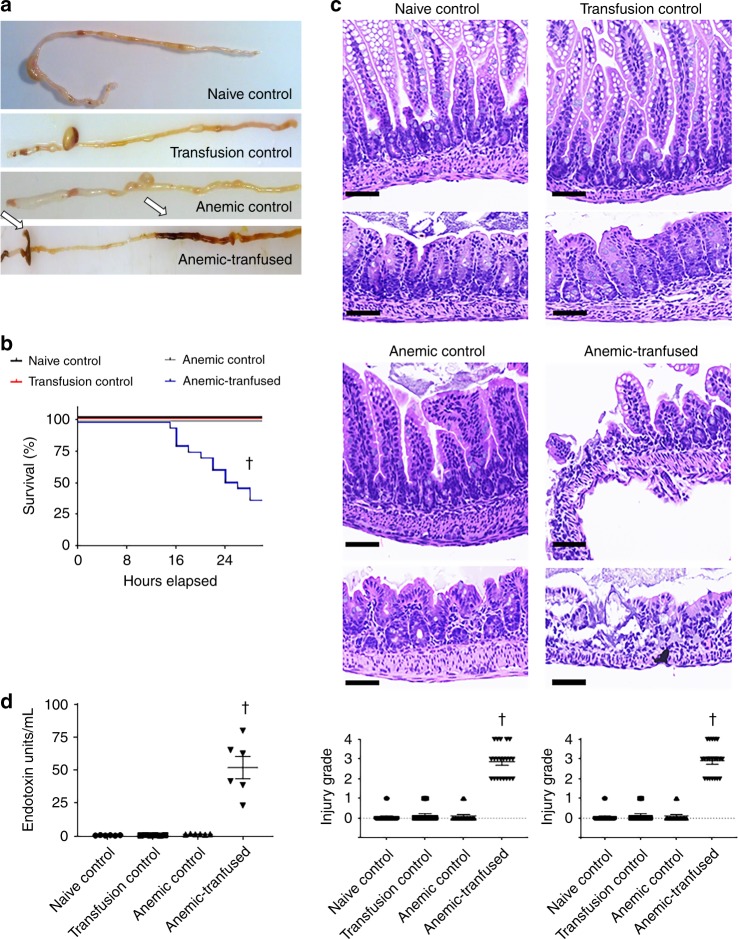
Red blood cell (RBC) transfusions trigger necrotizing enterocolitis (NEC)-like injury in anemic pups. **a** Representative photographs show bowel injury in ileocecal and mid-colonic regions (arrows) in anemic-transfused animals. Naive control, transfusion control, and anemic control did not develop bowel injury. **b** Kaplan–Meier curves summarize survival without intestinal injury; Mantel–Cox log-rank test, ^†^*P* < 0.001. **c** Representative photomicrographs (hematoxylin–eosin; magnification ×250) of ileum (left) and colon (right) show NEC-like injury in anemic-transfused mice. Scale bars = 100 µm. Scatter plots summarize severity of bowel injury. *N* = 42 animals in naive control, 21 in transfusion control, 18 in anemic control, and 21 in anemic-transfused groups. **d** Scatter plots show plasma lipopolysaccharide (LPS) levels in the four groups; *N* = 6 mice per group; Kruskal–Wallis *H* test with Dunn’s post test, ^†^*P* < 0.001 vs. naive control. All scatter plots summarize the data as means ± standard error of the mean

Anemic-transfused mice developed intestinal injury between 18 and 28 h after transfusion (Fig. 2a–c). In contrast, RBC transfusions caused no harm in transfusion controls, which had a normal hematocrit prior to the transfusion. Transfusion-associated bowel injury was marked by coagulative necrosis, inflammation, submucosal edema/separation, and interstitial hemorrhages, and thus resembled human NEC^37^. To compare the histopathological severity of intestinal injury, we used a 5-point scale (Supplementary Fig. 2a) that combined elements from other rodent models of NEC^38–40^ to describe both mucosal and submucosal changes. Consistent with the development of intestinal injury, the anemic-transfused mice showed evidence of bacterial translocation with elevated plasma lipopolysaccharide levels (Fig. 2d).

To ascertain that the bowel injury in anemic-transfused mice was not an artifact related to the introduction of *Serratia* into the gut microbiome, we compared pups colonized with *Serratia* vs. controls for the severity of bowel injury, and found no difference (Supplementary Fig. 2b). Furthermore, naive and transfusion controls that had been inoculated with *Serratia* showed no histological evidence of intestinal inflammation.

### Inflammation in the anemic and anemic-transfused intestine

In the neonatal intestine, autolytic changes resembling necrotic injury can set in within minutes after death. Therefore, we first confirmed that the crypt-villus disruption we observed in the anemic-transfused intestine occurred ante mortem and reflected NEC-like injury. Tissue sections were screened, and found negative, for signs of autolysis such as RBC hemolysis and loss of differential staining in the layers of the bowel wall. Consistent with this information, the anemic-transfused mice showed a 4-fold elevation (vs. control) in circulating fatty acid-binding protein 2 (FABP2), a cytosolic protein of enterocytes that is a sensitive marker of gut mucosal injury^41^.

**Fig. 3:**
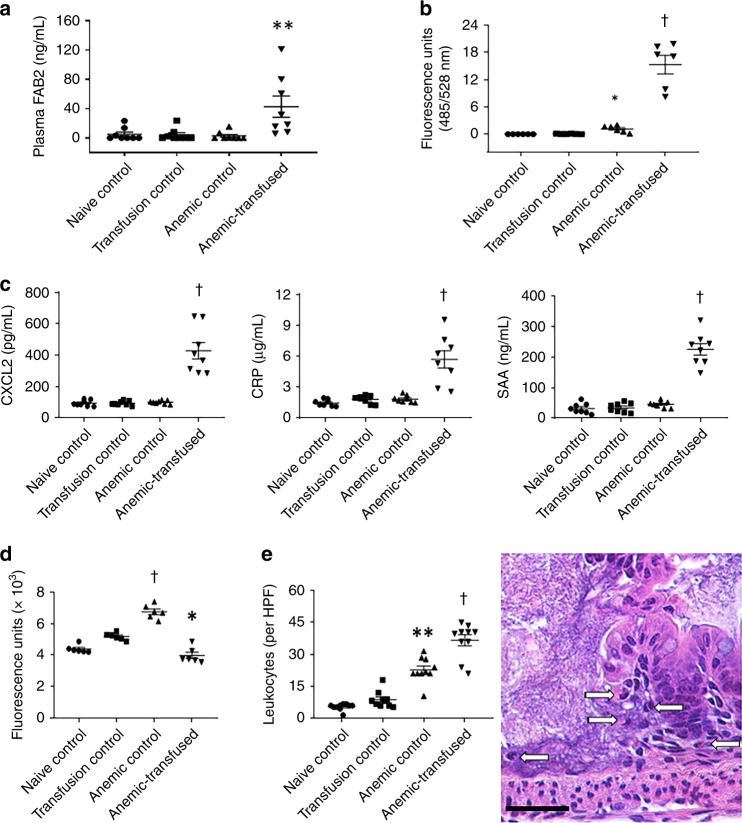
Inflammation in the anemic and anemic-transfused intestine. **a** Scatter column plots summarize plasma fatty acid-binding protein 2 (FABP2) concentrations. *N* = 8 mice per group. **b** Scatter plots show fluorescence readings in plasma 4 h after gavage with fluorescein isothiocyanate (FITC)-dextran. *N* = 6 mice per group. **c** Scatter plots show plasma CXC-motif ligand 2 (CXCL2), C-reactive protein, and serum amyloid A, measured by enzyme-linked immunosorbent assay (ELISA). *N* = 8 mice per group. **d** Seeding density of fluorescence-labeled polystyrene microspheres in ileocecal tissue. Scatter plots show fluorescence readings in tissue lysates, which provide an indirect estimate of regional blood flow. *N* = 6 mice per group. **e** Cellular inflammatory response in ileum and colon with necrotizing enterocolitis (NEC)-like injury. Scatter plots show number of leukocytes per high-magnification field. *N* = 10 mice per group. Right: Photomicrograph (hematoxylin–eosin, magnification ×250) of anemic-transfused colon shows numerous macrophages (arrows) at the edge of an ulcer. Scale bar = 100 µm. Data represent *N* = 10 anemic-transfused mice. Groups analyzed by Kruskal–Wallis *H* test with Dunn’s post test, *P < 0.05, **P < 0.01, ^†^*P* < 0.001 *vs*. naive control. All scatter plots summarize the data as means ± standard error of the mean

We evaluated gut barrier function by administering fluorescein isothiocyanate (FITC)-dextran by gavage in these mice 4 h prior to sacrifice and measured the FITC fluorescence signal in plasma just prior to sacrifice^42^. The anemic-transfused group showed increased plasma fluorescence readings (Fig. 3b). There was also a small, albeit statistically significant increase in intestinal permeability in anemic mice. In support of these data, the anemic and anemic-transfused intestine showed decreased epithelial immunoreactivity for the tight junction proteins, zonula occludens-1 (ZO-1) and occludin (Supplementary Fig. 3a).

Seeking further evidence that the bowel injury we observed in the anemic-transfused mice resembled human NEC, we measured plasma concentrations of CXC-motif ligand 2 (Cxcl2), C-reactive protein, and serum amyloid A, three inflammatory markers that are consistently increased in human NEC^43^. Compared to the control groups, the anemic-transfused mice showed elevated levels of these analytes (Fig. 3c).

To investigate the mechanism(s) by which transfusions caused NEC-like injury, we next asked whether transfused RBCs could impair intestinal perfusion and cause ischemic injury in a background of severe anemia, which presumably could have caused tissue hypoxia. To evaluate regional blood flow, we administered fluorescence-labeled polystyrene microspheres intravenously 1 h prior to sacrifice (schematic in Fig. 1a). These microspheres are lodged in capillaries, and the seeding density in tissues provides an indirect, but reliable estimate of regional perfusion^44^. We focused on the ileocecal area, which was affected in the anemic-transfused mice and is also frequently involved in human NEC. Compared to naive controls, there was increased microsphere seeding in anemic controls, but not in transfusion controls or the anemic-transfused mice (Fig. 3d; photomicrographs shown in Supplementary Fig. 3b). Similar, but less pronounced, changes in microsphere seeding were seen in other intestinal regions (Supplementary Fig. 3c). Lacking convincing support for bowel ischemia following RBC transfusions in transfusion controls and anemic-transfused mice, we focused on inflammation as an alternative mechanism of bowel injury. Our histopathological images of the anemic and anemic-transfused intestines (Fig. 2c) showed leukocyte infiltration with abundant macrophages (abundant cytoplasm, eccentrically placed reniform nucleus; Fig. 3e). Therefore, we next sought to define these macrophage populations.

### Macrophage infiltration in RBC transfusion-associated NEC

**Fig. 4:**
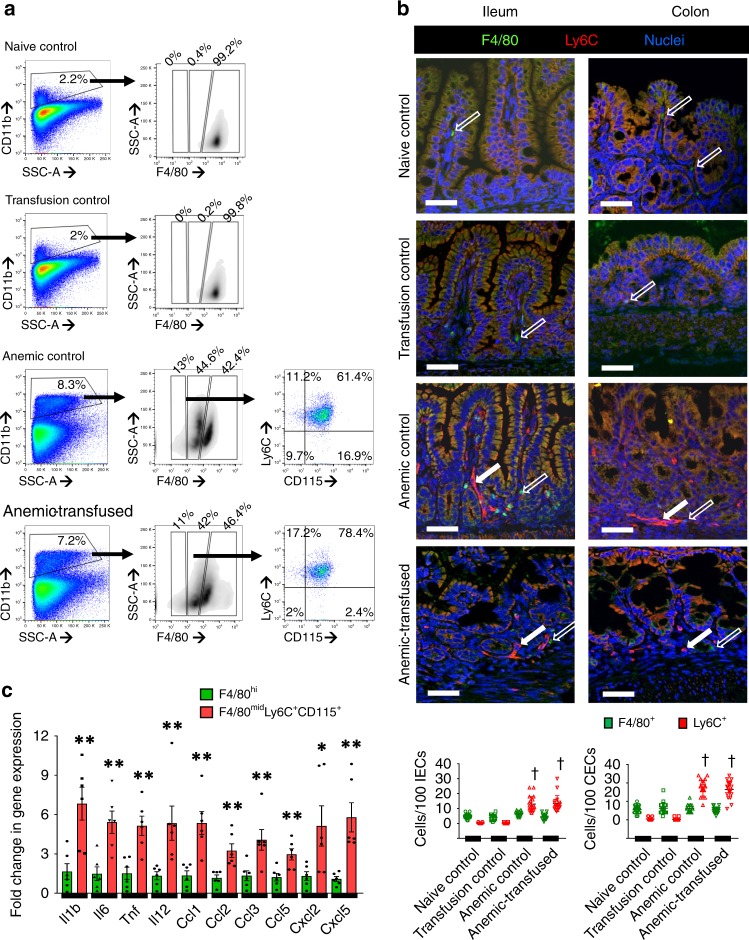
Macrophage infiltration in red blood cell (RBC) transfusion-associated necrotizing enterocolitis (NEC). **a** Representative scatter plots from control and anemic-transfused mice. The CD11b (+) myeloid cell fraction was enriched in anemic and anemic-transfused mice, and clustered into discrete F4/80 (hi) and F4/80 (mid) populations. The F4/80 (mid) macrophage population in anemic and anemic-transfused mice was CD115 (+) and Ly6C (+). **b** Fluorescence photomicrographs (ileum, colon; magnification ×800) showed scattered F4/80 (+) macrophages (green, open arrows) in naive and transfusion control. Numerous Ly6C (+) cells (red, solid arrows) were also seen in anemic and anemic-transfused intestine. Scale bar =50 µm. Scatter plots below summarize the number of Ly6C (+) and F4/80 (+) cells in each group. Kruskal–Wallis *H* test with Dunn’s post test, ^†^*P* < 0.001 vs. naive control. Data represent 10 mice per group. **c** Scatter bar diagrams summarize LPS-stimulated inflammatory cytokine expression in the F4/80 (hi) and F4/80 (mid) cell populations in vitro, measured by reverse transcriptase-quantitative polymerase chain reaction (RT-qPCR). *N* = 8 mice per group; Mann–Whitney *U* test, *P < 0.05, **P < 0.01

To define the cellular inflammatory response in the anemic and anemic-transfused intestine, we examined cell suspensions from enzymatically digested tissue samples by flow cytometry (Supplementary Fig. 4a). To differentiate inflammatory monocytes from neutrophils, CD11b^+^ myeloid cells were gated on macrophage markers^45^. In naive and transfusion control, the CD11b^+^ fraction was comprised of a single, F4/80^hi^-resident macrophage population. The anemic and anemic-transfused intestines showed enrichment of the CD11b^+^ fraction with an additional F4/80^mid^ CD115^+^ Ly6C^+^ cell population that resembled “inflammatory” macrophages derived from newly recruited blood monocytes (Fig. 4a; statistical analysis in Supplementary Fig. 4b). These findings were confirmed by immunofluorescence imaging, which showed resident macrophages expressing F4/80 but no/minimal Ly6C in naive and transfusion controls, and an additional Ly6C^+^ population in the anemic and the anemic-transfused intestine (Fig. 4b). To evaluate the inflammatory properties of F4/80^hi^ and F4/80^mid^ macrophages, we isolated these cell populations from the anemic intestine by cell sorting and measured lipopolysaccharide (LPS)-induced cytokine expression by reverse transcriptase-quantitative polymerase chain reaction (RT-qPCR). The F4/80^mid^ cells showed higher cytokine expression than the F4/80^hi^-resident macrophages (Fig. 4c).

The anemic and anemic-transfused intestines also showed a minor F4/80^low^ population that was comprised of Ly6G^mid^ Ly6B^+^ late-lineage neutrophil precursors and a few mature Ly6G^hi^ Ly6B^+^ neutrophils^46^ (Supplementary Fig. 4c, d). Interestingly, there was no change in lymphocytes. In support of this absence of a lymphocyte response, there was no difference in bowel injury in anemic-transfused mice lacking the recombination-activating gene 1 (RAG1^−/−^) vs. their wild-type (WT) littermates (Supplementary Fig. 4e).

### RBC transfusions activate intestinal leukocytes

**Fig. 5:**
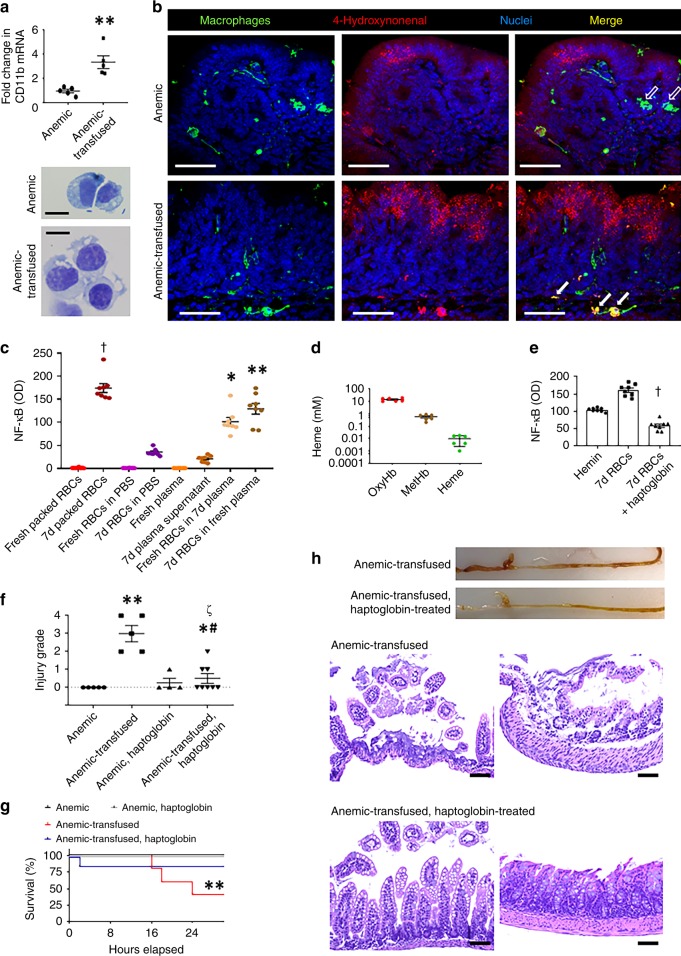
Red blood cell (RBC) transfusions activate intestinal leukocytes. **a** Scatter plots show CD11b expression in macrophages from anemic and anemic-transfused intestine; Mann–Whitney *U* test, **P < 0.01; photomicrographs (Wright–Giemsa; ×1000) below show “activated” morphology of macrophages from the anemic-transfused intestine. *N* = 5 mice per group. Scale bar =5 µm. **b** Fluorescence photomicrographs (magnification ×400) show immunoreactivity for 4-hydroxynonenal (4-HNE) in Ly6C (+) macrophages in anemic-transfused (solid arrows), but not in anemic control (open arrows). 4-HNE was also detected in nearby epithelium. Scale bar = 50 µm; *N* = 5 mice per group. **c** Scatter plots show relative nuclear factor-κB (NF-κB) activity (optical density) in NF-κB reporter RAW264.7 macrophages treated with fresh RBCs (control), 7-day RBCs, fresh RBCs resuspended in phosphate-buffered saline (PBS), 7-day RBCs in PBS, plasma supernatant from fresh and 7-day RBCs, fresh RBCs suspended in plasma from 7-day blood, and 7-day RBCs mixed with fresh plasma; *N* = 4 different donor mice, each with two technical replicates; Kruskal–Wallis *H* test, *P < 0.05, **P < 0.01, ^†^*P* < 0.001 vs. control. **d** Scatter plots show oxyhemoglobin (oxyHb), methemoglobin (metHb), and heme concentrations in supernatants from 7-day RBCs. *N* = 6 donor mice. **e** Scatter bar diagrams show NF-κB activity in reporter macrophages treated with 7-day RBCs vs. 7-day RBCs + haptoglobin (10 μg mL^−1^). *N* = 8 donor mice. Hemin was used as a positive control. ^†^*P* < 0.001 vs. 7-day RBCs, Mann–Whitney *U* test. **f** Scatter plots show severity of bowel injury in anemic-transfused pups pre-treated with haptoglobin. *N* = 5 anemic control, 4 haptoglobin-treated anemic control, and 8 each in anemic-transfused and haptoglobin-treated anemic-transfused; Kruskal–Wallis *H* test, *P < 0.05, **P < 0.01 vs. anemic control, ^#^*P* < 0.05 vs. haptoglobin-treated anemic, and ^ζ^*P* < 0.05 vs. anemic-transfused. **g** Kaplan–Meier curves summarize survival data from above experiment. One haptoglobin-treated mouse died 2 h after the procedure and was excluded; Mantel–Cox log-rank test, *P < 0.05. **h** Top: Representative photographs show intestinal injury in anemic-transfused mice, but not in haptoglobin-treated anemic-transfused mice. Bottom: Representative photomicrographs (hematoxylin–eosin; magnification ×250) of ileum (left) and colon (right) from these groups. *N* = 8 mice per group. Scale bars = 100 µm. All scatter plots summarize the data as means ± standard error of the mean

The number of F4/80^mid^ Ly6C^+^ macrophages in the anemic and the anemic-transfused intestine was similar, but bowel injury was seen only in the latter. Therefore, we next asked whether RBC transfusions could have activated the macrophages present in the anemic intestine. We isolated Ly6C^+^ cells from anemic control and the anemic-transfused intestine and compared CD11b expression by RT-qPCR. Consistent with our hypothesis of RBC transfusion-mediated macrophage activation, macrophages from the anemic-transfused intestine showed 3-fold higher CD11b expression than those from the anemic control. These macrophages from the anemic-transfused intestine also showed an “activated” appearance with increased ruffling and pseudopodia formation (Fig. 5a). To further define the activated state, we compared macrophages in the anemic control vs. the anemic-transfused intestine for evidence of reactive oxygen species (ROS) generation. Macrophages in the anemic-transfused, but not in the anemic intestine, showed 4-hydroxynonenal (4-HNE), which is a product of membrane lipid peroxidation^47^. 4-HNE was also detected in epithelial cells in close vicinity of these macrophages (Fig. 5b; intensity measurements and statistical analysis in Supplementary Fig. 5a) and in the few neutrophils present in the mucosa. Some neutrophils in the anemic-transfused intestine, but not in anemic control, displayed extracellular traps (Supplementary Fig. 5b), indicating that the effects of RBC transfusions were not specific for macrophages, but extended to all leukocytes present in the anemic intestine.

To determine the mechanism by which RBC transfusions activated gut macrophages, we first compared bowel injury in pups transfused with allogeneic (FVB donors) vs. syngeneic (C57BL/6 donors) RBCs^34^, but found no difference between the two groups (Supplementary Fig. 5c). Next, to ascertain the contribution of the RBC fraction vs. the plasma supernatants in transfused RBCs towards macrophage activation, we used a nuclear factor-κB (NF-κB) reporter murine RAW264.7 macrophage cell line (Imgenex, San Diego, CA). Using freshly isolated, leukoreduced RBCs as control, we compared 7-day-stored RBCs that we used for transfusions in our model, or the RBC or plasma fractions separated from these preparations; freshly isolated RBCs resuspended in plasma from 7-day RBCs; and 7-day RBCs resuspended in fresh plasma (Fig. 5c). Storage of RBC units increased the inflammatory potential of the transfused RBCs and the constituent RBC or plasma fractions (vs. fresh RBCs/plasma). Interestingly, fresh RBCs resuspended in plasma from 7-day RBCs, or 7-day RBCs suspended in fresh plasma had a comparable inflammatory effect. The presence of RBCs was necessary for macrophage activation; plasma fraction from 7-day stored RBCs caused only a minor increase in macrophage activation over freshly isolated plasma, which was significantly lower than the RBC fraction from 7-day RBC preparations used for transfusion. Based on these data, we postulated that the inflammatory effects of transfused RBCs were caused by factors released from stored RBCs such as free hemoglobin and/or heme. We next measured oxyhemoglobin (oxyHb), methemoglobin (metHb), and heme in our 7-day stored RBC preparations by an established spectrophotometric method^48^ and found high levels of cell-free oxyHb (Fig. 5d).

To further investigate the role of the plasma vs. the RBC fractions in the pathogenesis of transfusion-associated NEC-like injury, we administered plasma fractions from 7-day stored RBC preparations in naive controls and anemic controls (20 mL kg^−1^; *n* = 6 mice per group). These mice remained asymptomatic and did not develop bowel injury. The absence of bowel injury following the infusion of the plasma supernatants from 7-day stored RBC preparations suggested that the total amount of RBC degradation products such as free hemoglobin delivered through these plasma infusions was not sufficient, but that ongoing release of these products from the transfused RBCs in vivo was important in the pathogenesis of NEC.

To estimate the contribution of free hemoglobin to macrophage activation, we treated our NF-κB reporter macrophages with 7-day stored RBCs, and in some experiments, added recombinant haptoglobin, which is a natural chelator of free hemoglobin^49^. The addition of haptoglobin reversed the effect of the RBCs prepared for transfusions, on macrophage activation (Fig. 5e). These findings were of interest because human neonates have low plasma haptoglobin levels^49^, and our mouse pups also showed lower plasma haptoglobin than adult mice (Supplementary Fig. 5d). Therefore, we next asked whether intravenous administration of recombinant haptoglobin in anemic pups prior to transfusion could prevent NEC-like injury. As shown in Fig. 5f–h, haptoglobin pre-treatment was protective.

### RBC transfusions cause intestinal injury by activating TLR4

**Fig. 6:**
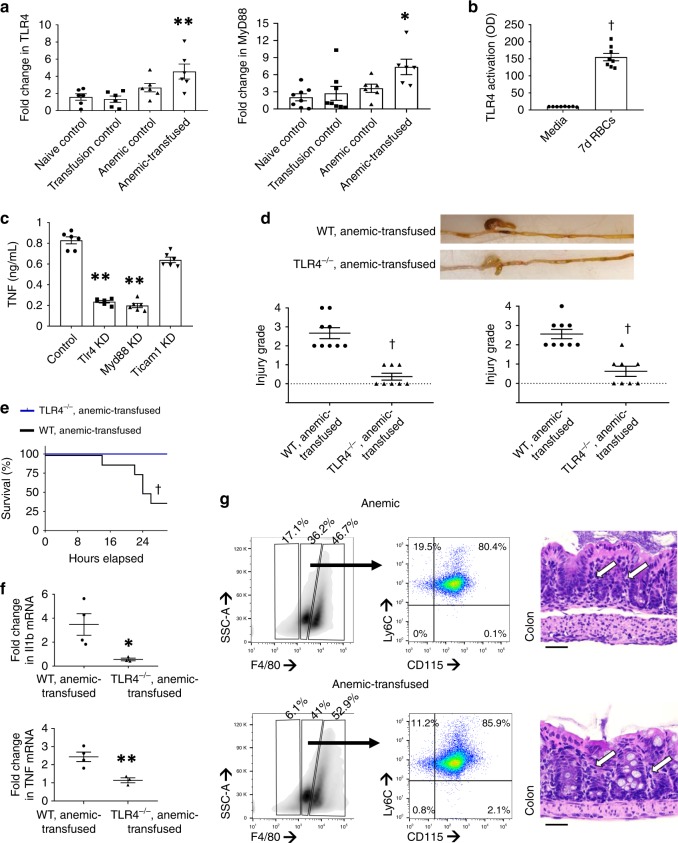
Red blood cell (RBC) transfusions cause intestinal injury by activating Toll-like receptor-4 (TLR4). **a** Scatter bar diagrams show fold change in Tlr4 and myeloid differentiation primary response gene 88 (Myd88) mRNA expression in the intestinal tissue from control and anemic-transfused mice. Kruskal–Wallis *H* test with Dunn’s post test, *P < 0.05, **P < 0.01 vs. naive control. *N* = 8 mice per group. **b** RBCs used for transfusion activate TLR4. Scatter bar diagram shows the activation of a reporter gene (secreted embryonic alkaline phosphatase) upon stimulation with 7-day-stored RBCs in TLR4-reporter cells. Mann–Whitney *U* test, ^†^*P* < 0.001 vs. naive control. *N* *=* 8 different donor mice. **c** RBCs used for transfusion fail to stimulate TNF production in RAW264.7 macrophages with Tlr4 and Myd88 knockdown. Ticam1 knockdown did not block TNF production. Kruskal–Wallis *H* test with Dunn’s post test, **P < 0.01 vs. control. *N* = 8 independent biological replicates per group. **d** Scatter plots show severity of transfusion-associated bowel injury in anemic-transfused Tlr4 (−/−) pups vs. wild-type (WT) littermates, in the ileum (left) and colon (right). Representative photographs above show ileal and colonic injury in WT, but not in the Tlr4 (−/−) intestine. *N* = 8 Tlr4 (−/−), 9 WT mice; Mann–Whitney *U* test, **P < 0.01, ^†^*P* < 0.001 vs. WT; **e** Kaplan–Meier curves summarize survival without intestinal injury in anemic-transfused Tlr4 (−/−) pups vs. WT littermates; Mantel–Cox log-rank test, ^†^*P* < 0.001. **f** Scatter plots summarize Il1b (top) and Tnf (bottom) expression in ileum from anemic-transfused Tlr4 (−/−) pups vs. WT littermates, expressed as fold change over naive control of the same genotype. *N* = 3 Tlr4 (−/−), 4 WT mice; Mann–Whitney *U* test, *P < 0.05, **P < 0.01. **g** Representative flow cytometry scatter plots of the CD11b-positive myeloid cell fraction in the anemic and anemic-transfused Tlr4 (−/−) intestine show a F4/80 (mid) CD115 (+) Ly6C (+) cell population. Hematoxylin and eosin (H&E)-stained photomicrographs of the colon from anemic and anemic-transfused Tlr4 (−/−) mice show increased cellularity (arrows) in the lamina propria. Scale bars = 50 µm. *N* = 8 Tlr4 (−/−), 9 WT mice. All scatter plots summarize the data as means ± standard error of the mean

We next sought the molecular mechanism(s) by which RBC transfusions caused intestinal injury. Based on emerging information that hemoglobin may serve as an endogenous ligand for Toll-like receptor-4 (Tlr4)^50,51^, we measured gene expression of major signaling mediators in TLR4-activated and downstream NF-κB pathways and inflammatory cytokines by RT-qPCR. The anemic-transfused intestine showed increased expression of Tlr4 and the myeloid differentiation primary response gene 88 (Myd88; Fig. 6a). NEC-like injury also induced interleukin-1β (Il1b), tumor necrosis factor (Tnf), monocyte chemokines CC-motif ligand (Ccl)-2, Ccl3, Ccl5, Cxcl5, Nfkb1, and the reticuloendotheliosis proto-oncogene, NF-κB subunit (Rel/Crel). The anemic intestine showed increased Tnf, Myd88, and Rel expression (Supplementary Fig. 6a). Direct confirmation of TLR4 activation by transfused RBCs was obtained upon treatment of a specific TLR4-reporter cell line (HEK-blue-TLR4 cells; Invivogen) with 7-day RBCs (Fig. 6b). RNA knockdown of Tlr4 and Myd88, but not Ticam1 (toll-like receptor adaptor molecule 1) suppressed TNF production in RAW264.7 macrophages treated with 7-day RBCs (Fig. 6c).

We next evaluated intestinal injury in anemic-transfused TLR4^−/−^ [B6(Cg)-*Tlr4*^*tm1.2Karp*^/J] mice. In contrast to WT littermates, TLR4^−/−^ mice were protected against transfusion-associated bowel injury and related inflammation (Fig. 6d–f). Interestingly, the loss of TLR4 did not prevent infiltration with monocyte-derived macrophages in the anemic and anemic-transfused TLR4^−/−^ mice (Fig. 6g; statistical analysis in Supplementary Fig. 6b). The detection of newly recruited monocytes in the intestines of anemic and anemic-transfused TLR4^−/−^ mice, but no tissue damage, was consistent with our hypothesis that an additional activation step (provided by RBC transfusions) was needed for these cells to cause inflammation and bowel injury.

### Macrophages are required for RBC transfusion-associated NEC

**Fig. 7:**
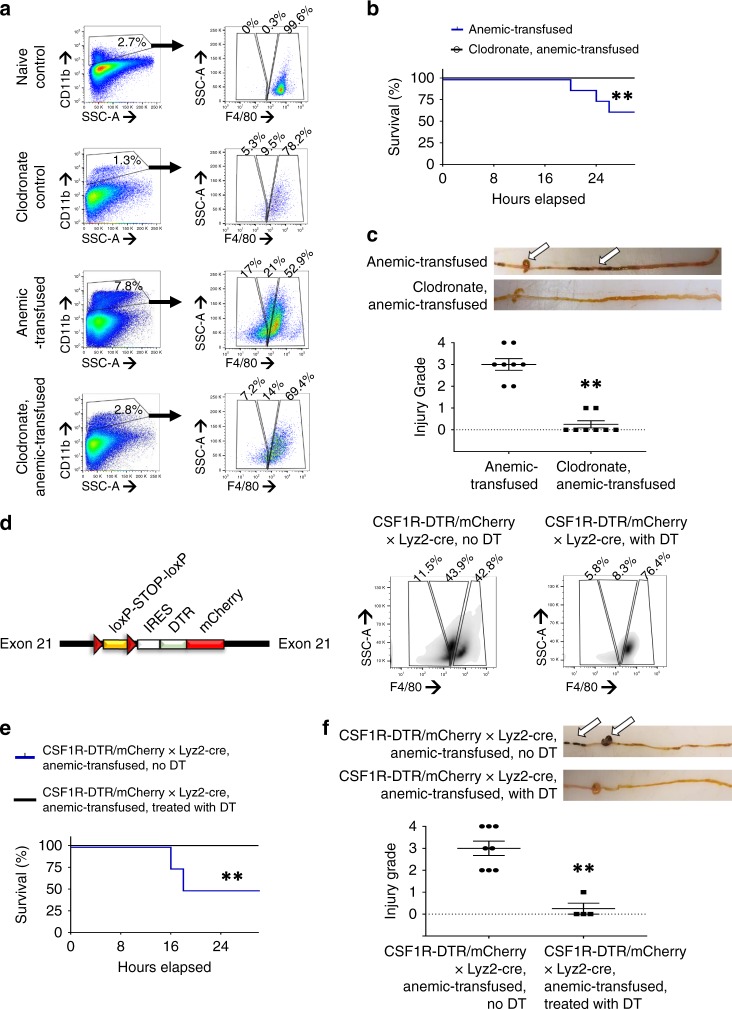
Macrophages are required for red blood cell (RBC) transfusion-associated necrotizing enterocolitis (NEC). **a** Representative scatter plots from naive control, anemic-transfused, and corresponding clodronate-treated groups show depletion of F4/80 (hi) macrophages in clodronate-treated naive control, and of both F4/80 (hi) and F4/80 (mid) populations in clodronate-treated anemic-transfused mice. *N* = 8 mice per group. **b** Kaplan–Meier curves show survival without intestinal injury in anemic-transfused vs. clodronate-treated anemic-transfused pups; *N* = 8 mice per group, Mantel–Cox log-rank test, **P < 0.01. **c** Top: Representative photographs show intestinal injury in an anemic-transfused pup (arrows) and the absence of injury in a clodronate-treated anemic-transfused pup; bottom: **d **Scatter plots show severity of bowel injury in anemic-transfused vs. clodronate-treated anemic-transfused mice. *N* = 8 mice per group; 2 mice in the clodronate-treated group died within 1 h of presumed procedure-related causes and were excluded; Mann–Whitney *U* test, *P < 0.05. **d** Schematic (left) of the bacterial artificial chromosome (BAC) encoding a diphtheria toxin receptor (DTR)-mCherry fusion protein preceded by a loxP-flanked transcriptional Stop element, driven by the CD115 promoter in C57BL/6-Tg(Csf1r-HBEGF/mCherry)1Mnz/J mice; IRES = internal ribosome entry site. Right: representative scatter plots from anemic CSF1R-DTR/mCherry × Lyz2 (lysozyme 2)-cre mice show depletion of F4/80 (mid) CD115 (+) Ly6C^hi^ macrophages following DT administration. **e** Kaplan–Meier curves below show survival without intestinal injury of mock- and DT-treated anemic-transfused mice; *N* = 6 mock-treated, 4 DT-treated anemic-transfused mice, Mantel–Cox log-rank test, **P < 0.01. **f** Top: Representative photographs show intestinal injury in anemic-transfused (arrows) vs. the absence of injury in a DT-treated, anemic-transfused pup. Bottom: Scatter plots show severity of bowel injury in the mock- vs. DT-treated anemic-transfused CSF1R-DTR/mCherry × Lyz2-cre mice. *N* = 6 mock-treated, 4 DT-treated anemic-transfused mice; Mann–Whitney *U* test, **P < 0.01. All scatter plots summarize the data as means ± standard error of the mean

To ascertain the causative role of macrophages, we treated some normal and anemic mouse pups with clodronate liposomes on P8 to deplete intestinal macrophages (Fig. 7a; Supplementary Fig. 7a). These macrophage-depleted mice were protected against intestinal injury following subsequent transfusions on P11 (Fig. 7b, c).

To investigate the specific role of monocyte-derived inflammatory macrophages, we used transgenic mice [C57BL/6-Tg(Csf1r-HBEGF/mCherry)1Mnz/J] that carry a bacterial artificial chromosome encoding a CD115 promoter-driven diphtheria toxin receptor-mCherry (DTR-mCherry) fusion protein, which is preceded by a loxP-flanked transcriptional Stop element (Fig. 7d). Crossing these animals with Lyz2 (lysozyme 2)-cre mice deletes the floxed Stop element and allows expression of DTR-mCherry in inflammatory monocytes. In these pups, administration of diphtheria toxin (DT, 5 ng g^−1^ weight) selectively depleted the inflammatory F4/80^mid^ CD115^+^ Ly6C^hi^ macrophages but not the resident macrophages (Fig. 7d, Supplementary Fig. 7b, c). Similar to our studies with clodronate liposomes, this selective depletion of inflammatory macrophages was also protective (Fig. 7e–g).

### Macrophages are activated during transfusion-associated NEC

**Fig. 8:**
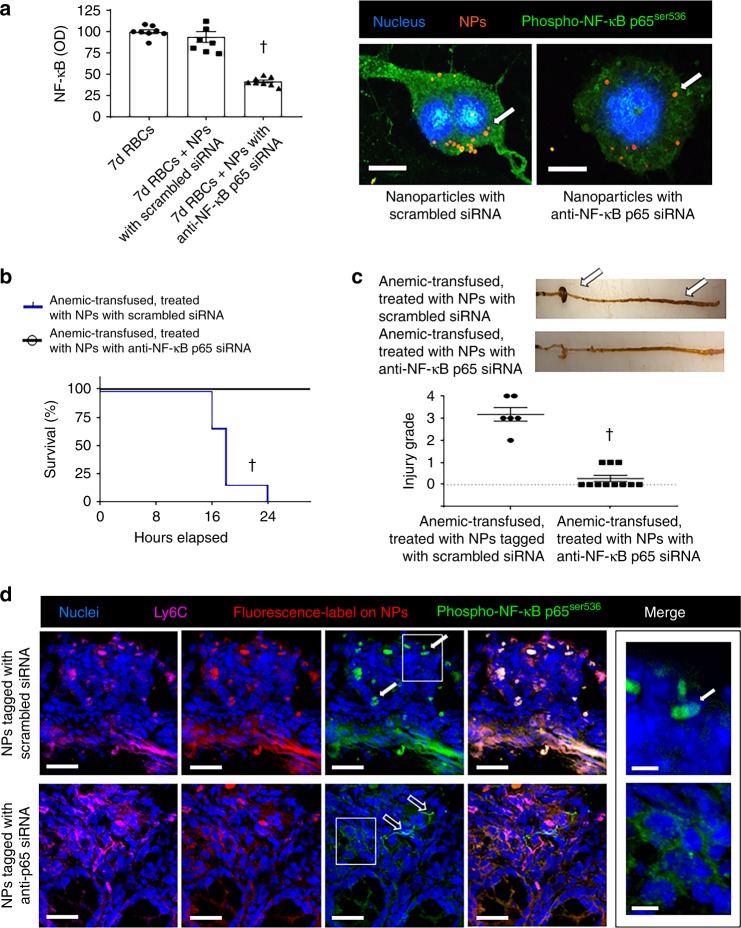
Macrophages are activated during transfusion-associated necrotizing enterocolitis (NEC). **a** Scatter bar diagrams show nuclear factor-κB (NF-κB) activity in reporter cells treated with nanoparticles (NPs) containing small interfering RNA (siRNA) against NF-κB p65 for 24 h, followed by 7-day-stored red blood cell (RBCs). Controls included no NPs or NPs containing scrambled siRNA; *N* = 8 donors; Kruskal–Wallis *H* test with Dunn’s post test, ^†^*P* < 0.001. Fluorescence photomicrographs (magnification ×2400) show “activated” appearance and nuclear translocation of phospho-NF-κB p65 (green) in a macrophage treated with control NPs. Cells treated with anti-p65 NPs did not show phospho-NF-κB p65. Arrows indicate NPs. Scale bar = 5 µm. **b** Kaplan–Meier curves show survival without intestinal injury in anemic-transfused pups treated with control or anti-p65 NPs. NPs with anti-p65 siRNA were protective; Mantel–Cox log-rank test, ^†^*P* < 0.001. **c** Photographs show intestinal injury in an anemic-transfused pup treated with control NPs (arrows), but no injury in a pup treated with NPs containing anti-p65 siRNA. Scatter plots below summarize the severity of bowel injury in the two groups. *N* = 6 anemic-transfused mice treated with control NPs, 11 anemic-transfused treated with anti-p65 NPs; Mann–Whitney *U* test, ^†^*P* < 0.001. **d** Fluorescence photomicrographs (magnification ×400) of proximal colon from anemic-transfused mice treated with either control or anti-p65 NPs show immunoreactivity for Ly6C (magenta), fluorescence tag on NPs (red), and phospho-NF-κB p65 (green). Mice treated with control NPs show tissue damage and nuclear translocation of phospho-p65, whereas those treated with anti-p65 NPs show preserved histoarchitecture and no immunoreactivity for phospho-p65 in Ly6C^+^ macrophages. Cytoplasmic staining for phospho-p65 was seen in two cells (open arrows) that did not express Ly6C. Scale bar = 50 µm. High-magnification photomicrographs on right (×2000; phospho-p65 staining in area in boxes) highlight nuclear localization (arrow) of phospho-p65 in the control NP group; *N* = 5 mice per group. All scatter plots summarize the data as means ± standard error of the mean

Seeking additional evidence that macrophages present in the anemic intestine needed to undergo inflammatory activation to cause bowel injury, we next used an established, nanoparticle (NP)-based approach to block the NF-κB pathway in macrophages in vivo^52^. These NPs are comprised of a fluorescence-tagged small interfering RNA (siRNA) against the NF-κB p65 transcript, complexed with a melittin (principal component of bee venom)-derived cationic amphipathic peptide that improves siRNA delivery by initiating endosomal escape. These NPs are preferentially phagocytosed by immune cells at sites of inflammation^52^. In preliminary studies, NPs containing anti-p65 siRNA blocked NF-κB activation in our reporter macrophages in vitro following stimulation with 7-day-stored RBCs (Fig. 8a). We next administered these anti-p65 or control (scrambled siRNA) NPs in anemic mice (1 nmol intravenously and 2 nmol intraperitoneal; pre-determined optimum) 24 h prior to RBC transfusion. Mice treated with anti-p65 NPs were protected (Fig. 8b, c). The presence of these NPs inside the Ly6C^+^ intestinal macrophages was evident from detection of the Cy3 fluorescence tag of our NPs in these cells by fluorescence microscopy. Mice treated with control NPs showed nuclear translocation of phosphorylated NF-κB p65 in their Ly6C^+^ intestinal macrophages. In contrast, animals treated with anti-p65 NPs showed no p65 phosphorylation or nuclear translocation (Fig. 8d).

### Clinical variables relevant in transfusion-associated NEC

**Fig. 9:**
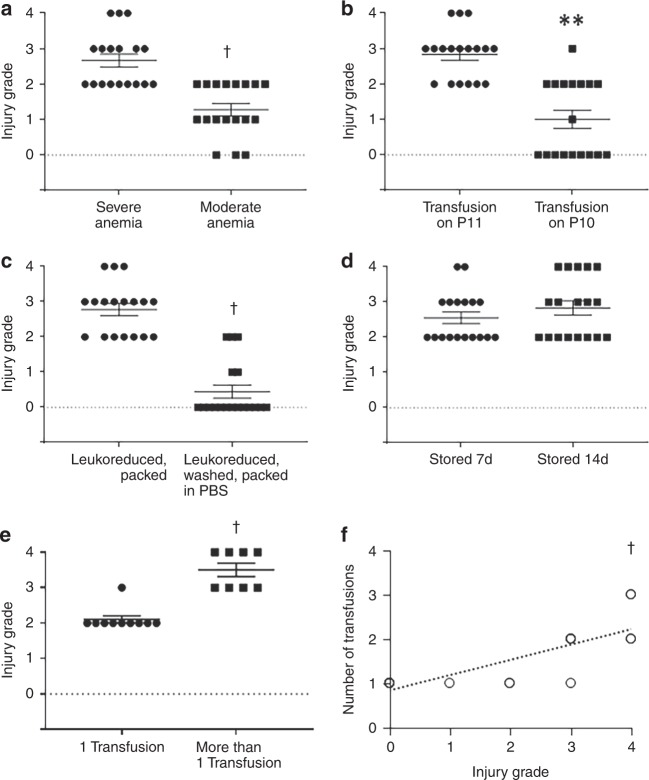
Clinical variables relevant in transfusion-associated necrotizing enterocolitis (NEC). Scatter plots show severity of NEC-like injury (means ± standard error) following: **a** red blood cell (RBC) transfusions in mice with severe (hematocrit 20–24%) vs. moderate anemia (hematocrit 25–30%); **b** transfusions administered soon after the hematocrit dropped to 20–24% (on P10) vs. 24 h later (P11); **c** transfusions with leukoreduced, packed RBCs vs. RBCs that were leukoreduced, washed, and resuspended in phosphate-buffered saline (PBS) before storage; **d** transfusions with RBCs stored for 7 vs. 14 days; **e** single vs. multiple RBC transfusions. *N* = 18 mice per group in all above experiments; Mann–Whitney *U* test, **P < 0.01, ^†^*P* < 0.001. **f** Scatter plot shows positive correlation (Spearman’s *r* = 0.865) between number of transfusions and severity of injury; ^†^*P* < 0.001. All error bars show standard error of the mean

We next used our murine model to investigate whether the severity of anemia prior to transfusion affects transfusion-associated NEC-like injury. Mouse pups with severe anemia (hematocrit 20–24%) developed more severe bowel injury as compared to those with moderate anemia (hematocrit 25–30%; Fig. 9a). We next asked whether the time spent in a severely anemic state prior to transfusion affected NEC-like injury. Mice transfused on P10 (soon after the last phlebotomy) sustained less bowel damage than those transfused 24 h later on P11 (Fig. 9b).

In most experiments, we transfused mice with packed, leukoreduced RBCs. However, in view of the inflammatory effects of soluble factors present in the plasma supernatant from stored RBCs (Fig. 5a), we asked if removal of the plasma fraction prior to storage would be protective. Indeed, when RBCs were leukoreduced, washed, and resuspended in phosphate-buffered saline (PBS) prior to storage, the severity of bowel injury was decreased (Fig. 9c). Increasing duration of RBC storage (7 days vs. 14 days) did not affect bowel injury (Fig. 9d).

Finally, we investigated whether multiple RBC transfusions increased the risk of bowel injury. In our experimental protocol, we measured the hematocrit on P12 and P13, and if the hematocrit was <30%, we transfused these pups again with 20 mL kg^−1^ RBCs aiming to achieve a hematocrit above 35%. Pups that received multiple transfusions showed more severe injury (Fig. 9e), and the severity of bowel injury showed a significant correlation with the total number of RBC transfusions (Spearman’s *r* = 0.865, *P* < 0.001; Fig. 9f).

## Discussion

We present a detailed investigation of RBC transfusion-associated NEC and its mechanisms. RBC transfusions triggered NEC-like bowel injury in anemic mouse pups, but were not injurious when administered in mice with normal hematocrit. Severe anemia caused a low-grade inflammatory state in the intestinal mucosa with macrophage infiltration, and subsequent RBC transfusions activated these cells via a TLR4-mediated mechanism to cause bowel injury. Consistent with clinical observations^10,19^, the severity and the duration of severe anemia prior to the RBC transfusion were important predictors of transfusion-associated bowel injury. The onset of NEC-like injury between 18 and 28 h after RBC transfusions was also consistent with the typical course of transfusion-associated NEC in human infants.

Our murine model of transfusion-associated NEC resembled human NEC in its regional predilection for the ileocecal and mid-colonic segments, histopathological findings of coagulation necrosis and macrophage infiltration, circulating markers such as Cxcl2, C-reactive protein, and serum amyloid A, and the induction of inflammatory genes in affected tissues^43,53^. Interestingly, NEC-like bowel injury can be induced in rodents through a variety of exposures, such as subjecting pups to repeated hypoxia and hypothermia, enteral administration of trinitrobenzene sulfonate as a non-specific immunological stimulant, or by chemical ablation of Paneth cells^38,40,54^. The shared pathoanatomy of these diverse models lends credence to the possibility that NEC represents the generic injury response of the intestine during a particular developmental epoch and not necessarily the specific response to a temporally-proximate causal trigger^38,53^.

The detection of macrophage infiltration in severe anemia is interesting. Following transfusion, RBC degradation products activated these macrophages, and, in turn, these cells produced secondary mediators such as ROS and inflammatory cytokines, leading to tissue damage. The central, deleterious role of macrophages in transfusion-related injury was evident from the protective effects of macrophage depletion. Similar protective effects were observed when macrophage activation was blocked by the administration of anti-NF-κNPs. Although the histopathology of transfusion-associated human NEC has not been described yet, macrophage infiltration and activation are well-documented in human pathological specimens of NEC^37,55,56^ and in experimental NEC models^38,57^. Recently, Managlia et al.^57^ showed that NF-κB activation in inflammatory monocyte-derived macrophages was required for the development of NEC-like injury in mice. However, the role of other leukocyte lineages is less consistent across NEC models. We did not detect a change in lymphocyte populations or a difference in intestinal injury between WT vs. RAG1^−/−^ mice. Our observations contrast with the findings of Egan et al*.*^58^, who showed that TLR4-mediated influx of T-helper type 17 lymphocytes was required for the development of NEC-like injury. To induce NEC, mouse pups were separated from the dam on P7–P8 and fed infant formula, colonized with bacterial flora from a human infant with NEC, and subjected to repeated exposures to hypoxia. In this model, RAG1^−/−^ mice were protected from NEC and transfer of intestinal lymphocytes from NEC mice into naive mice induced intestinal inflammation. Similarly, we also did not find any injury-related depletion of myeloid-derived suppressor cells (MDSCs). He et al.^59^ induced NEC in P1 mice by repeated exposure to hypoxia and hypothermia, and showed that the transitory presence of MDSCs was critical for protection against inflammatory intestinal injury. Our NEC model differs from Egan et al.^58^ and He et al*.*^59^ in the age of mice and the specific physiological stressors used to induce intestinal injury. Further study is needed to investigate whether these differences between NEC models in the cellular inflammatory response may indicate possible, yet unknown heterogeneity in human NEC related to the age of onset and specific risk factors.

In our model, free hemoglobin present in RBC transfusions activated macrophages via a TLR4-dependent mechanism. The pathogenetic role of free hemoglobin in macrophage activation and intestinal injury found support in the protective effect of recombinant haptoglobin. The role of TLR4 in NEC pathogenesis has been noted in other NEC models^60^, and our findings in RBC transfusion-associated bowel injury further highlight its importance as a unifying mechanism in NEC. We also investigated oxidative stress as an alternative mechanism for inflammation in the anemic-transfused intestine. In support, we detected lipid peroxidation in macrophages and nearby epithelial cells that could be explained by free hemoglobin-dependent redox cycling^61^. However, 4-HNE seemed to be differentially expressed with a more robust fluorescence signal in macrophages than in the epithelium, indicating that ROS production was specific to activated macrophages and may not represent a global oxidative stress due to RBC transfusion-related restoration of tissue oxygen delivery.

We used mice on P11–P13 to study transfusion effects in premature neonates. In terms of gut development, P13 mice were relevant because the murine intestine at birth resembles the midgestation human intestine and reaches the structural/functional maturity of the term human neonate (mucosal histoarchitecture, leukocyte populations, digestive enzymes, and transporters) only by P18–P21^38,62,63^. P11–P13 pups showed a baseline hematocrit of 40–50% that was comparable to human neonates, and the hematocrit dropped predictably with timed phlebotomy to 20–24% levels typical of severe neonatal anemia. Premature infants typically develop severe anemia either within the first few weeks due to phlebotomy losses, or later, beyond 4 weeks due to the classical anemia of prematurity, which is a multifactorial process related to hypoactivity of the immature bone marrow, insufficient erythropoietin production, high turnover of neonatal RBCs, rapid somatic growth, and nutritional deficiencies^64^. Timed phlebotomy is perhaps a more accurate representation of early-onset neonatal anemia, but its biological effects have also been validated to model anemia of prematurity^65^. In our study, anemic pups developed normocytic, hypochromic anemia, and low reticulocyte hemoglobin, indicating that incipient iron deficiency due to blood loss may also have contributed^64^. These hematological changes are frequently seen in convalescing premature infants^65^.

We transfused RBCs from adult FVB/NJ donors into C57BL/6 recipients, which is an established model of allogeneic transfusion^34^, at transfusion thresholds similar to those used in human premature infants^66^. Murine RBCs were prepared for transfusion in steps similar to human blood banking, including the use of citrate-phosphate-dextrose-adenine-1 (CPDA-1) anticoagulant, leukoreduction, and packing to 70% hematocrit. However, there were two important differences. First, we stored murine RBCs for only 7–14 days, whereas human RBCs are often stored for up to 42 days. Murine RBCs, particularly those from FVB/NJ mice, have a shorter shelf-life than human RBCs and deteriorate rapidly beyond 14 days^67^. Second, we achieved leukoreduction by removing the buffy coat and not by filtration methods used in blood banks. Although high-efficiency neonatal leukocyte reduction filters has been used for murine RBCs, even the smallest filters posed logistical difficulties such as the need to pool blood from 8 to 10 adult mice and loss of RBC viability^67^.

When administered in the absence of anemia, RBC transfusions caused no harm. The severity of transfusion-associated bowel injury was clearly related to the severity and duration of anemia, which are likely to have accentuated the inflammatory changes in the intestine and thus predisposed to transfusion-induced injury. These findings are relevant because RBC transfusions are often administered in premature infants for poorly understood signs such as poor weight gain or apnea–bradycardia events, and the risk stratification for the adverse effects of transfusions may need to factor in the baseline hematocrit. Mechanistically, there was small but significant increase in mucosal permeability in the anemic control, and further study is needed to determine whether this leakiness could lead to bacterial translocation and inflammation. Interestingly, the macrophage infiltration we detected in the anemic intestine remained intact in anemic TLR4^−/−^ mice and in anemic mice that carried few native Gammaproteobacteria and did not receive *Serratia*. Although Gram-negative bacteria can be recognized via pathogen recognition receptors other than TLR4 and may still play a role, these findings are of interest in light of emerging data on enteric dysbiosis in NEC pathogenesis^30^, and call for investigation to ascertain if these microbial communities also play a role in the subgroup of infants who develop NEC following transfusions.

We did not detect an effect of RBC storage age on intestinal injury, but found washing of RBCs at storage to be protective. Washing can reduce inflammatory lipids, microparticles, and cytokines, but these findings need further examination before any clinical recommendations because concerns remain about the effects of washing on RBC viability^68^. In our model, mice receiving multiple RBC transfusions sustained more severe injury. We administered repeat transfusions in pups that failed to show a rise in hematocrit after the first transfusion. The likely explanation for persistent anemia in these mice is that intestinal injury may have occurred after the very first transfusion, and may have caused occult blood loss either in the injured bowel or to hemolysis^69^. However, the possibility of a dose effect where repeat transfusions may disproportionately increase the risk of NEC, also needs consideration.

Our NEC model involved several potentially stressful interventions such as phlebotomy, intravenous RBC transfusions, gavage, and intraperitoneal injections. Stress can trigger a variety of physiological responses and can even accentuate tissue injury^70^. We controlled for two exposures, phlebotomy-induced anemia and RBC transfusions, and the absence of bowel injury in these animals was reassuring. Maternal separation^71^ was not a concern as pups were housed with and nursed by the dam throughout the study period. We also controlled for gavage and intraperitoneal injections. However, we did not have controls matched for dual exposure to anemia and transfusions. We contemplated including, but eventually decided not to use biochemical stress markers such as cortisol because of the anticipated confounding effect of bowel injury. The effects of stress in premature infants remain a subject of investigation; emerging data link stress with adverse neurodevelopmental outcomes^72^, but the association with NEC remains unclear.

Current information on the RBC transfusion-associated NEC is largely limited to retrospective case–control studies^2–24^. The association finds biological plausibility in observations of altered splanchnic autoregulation following RBC transfusions in premature infants^22,73^, but several systematic reviews and meta-analysis of observational data have also emphasized the inconsistency in these findings^4,25,26^. A recent, large prospective study^10^ detected an association of NEC with anemia, but not with RBC transfusions. Some of this uncertainty in clinical studies may be explained by our findings that neither anemia nor RBC transfusions are independently sufficient as causes of bowel injury, but that the occurrence of NEC requires sequential exposure to both anemia and transfusions. Interestingly, the risk and severity of bowel injury increased in our model with decreasing pre-transfusion hematocrit. These findings call for re-evaluation of current transfusion guidelines for premature infants; the adoption of restrictive transfusion guidelines has reduced the frequency of RBC transfusions in these patients, but may have inadvertently increased the risk of complications such as NEC.

## Methods

### Experimental design

Animal studies were performed after ethical approval from the Institutional Animal Care and Use Committees at the University of South Florida and Johns Hopkins University, and complied with all relevant ethical regulations for animal testing and research. C57BL/6 mice from each litter were randomly assigned to four study groups: (a) naive control; (b) transfusion control; (c) anemic control; and (d) anemic-transfused mice. Sample size was estimated for intestinal injury at *α* = 0.05 and 80% power (Lehmann’s method for non-Gaussian data). P7 mice were inoculated with a clinical isolate of *Serratia marcescens* (10^4^ CFU, gavage) grown in nutrient agar/broth (American Type Culture Collection, Manassas, VA). Naive controls were maintained without intervention. Transfusion controls received an intravenous RBC transfusion (20 mL kg^−1^) into the retro-orbital venous plexus, injected in two aliquots on each side) on P11. RBCs for transfusion were collected in a sterile fashion from adult FVB/NJ donors after sacrifice into CPDA-1 (Sigma, St. Louis, MO; catalog #C4431; 6 parts blood:1 part CPDA-1). After centrifugation at 400 × *g* for 10 min, the buffy coat was removed along with some plasma and top part of the RBC layer for leukoreduction^36^ and the supernatant was reduced to achieve a hematocrit of 70% before storing at 4 °C in dark for 7–14 days. Transfused RBCs were confirmed to be free of endotoxin contamination before use (limulus lysate assay; Thermo Fisher, Waltham, MA; catalog #88282). Anemic controls were subjected to facial vein phlebotomy to collect 40 µL blood on P2, P4, P6, P8, and P10. Hematocrits and RBC indices were measured at each phlebotomy and then every day on P11–P13; 5 µL blood was diluted 1:20 in Cellpak reagent (Sysmex America, Mundelein, IL; catalog #DCL-310A), and analyzed in the Sysmex XT-2000iV veterinary hematology analyzer. Anemic-transfused mice underwent phlebotomy as above and then received a transfusion on P11. If hematocrit remained <30% on P12 or P13, the transfusions were repeated. Phlebotomy and intravenous injections can be technically challenging in P11–P13 mice, but in the hands of trained personnel, both procedures are well tolerated by the pups and success rates approach 100%. The use of surgical magnifier glasses is helpful.

Pups were housed with and nursed by the dam throughout the study period. These animals were monitored closely and weighed at regular intervals (Supplementary Table 2). Animals were sacrificed on P13, or earlier if they developed physical distress, which was typically due to bowel injury, using CO_2_ inhalation, and cervical dislocation. To measure gut barrier function, we administered FITC-dextran by gavage (10 kDa, 400 mg kg^−1^) 4 h before sacrifice and measured the FITC fluorescence signal in plasma samples obtained just prior to sacrifice^42^. To measure regional blood flow, 10^4^ fluorescence-labeled polystyrene microspheres were administered intravenously (15 μm; Thermo Fisher) 1 h before sacrifice and the fluorescence signal was measured in tissue lysates^44^. In some experiments, we transfused mice with moderate anemia (hematocrit 25–30%) or on P10 instead of P11, prepared RBCs by resuspending in phosphate-buffered saline before storage, or used RBCs from adult C57BL/6 (syngeneic) donors.

### Genetically modified mice

C57BL/6-Tg(Csf1r-HBEGF/mCherry)1Mnz/J (stock #024046), B6.129P2-Lyz2tm1(cre)Ifo/J (stock #004781), B6(Cg)-Tlr4tm1.2Karp/J (stock #029015), and B6.129S7-Rag1tm1Mom/J (stock #002216) were purchased from The Jackson Laboratory (Bar Harbor, ME) and used per vendor’s guidelines.

### Microbiome analysis

Total DNA was isolated from tissue samples harvested on P11 from 100 to 250 (DNeasy Blood and Tissue Kit; Qiagen, Carlsbad, CA; catalog #69504) and was used to amplify the V4 region of 16S ribosomal RNA (rRNA) gene using polymerase chain reaction with modified 515F and 806R primers. These DNA segments were sequenced using the MiSeq platform (Illumina, San Diego, CA) to generate about 15,000 paired-end reads of 250bp size per sample. Demultiplexed DNA sequences were then analyzed for bacterial identification to genus level using the CLC Biomedical Workbench 3.5.3 (Qiagen). Operational taxonomic units (OTUs) were assigned based on 97% sequence identity to the Greengenes v13.8 reference database, and their relative abundance (percentage) was computed. Bacterial diversity was measured within samples (α-diversity) as the number of OTUs, phylodiversity, and the Chao 1, Simpson, and Shannon indices; between-samples (β-diversity) by principal coordinate analysis and permutational multivariate analysis of variance (PERMANOVA) of weighted and unweighted UniFrac distance matrices, Jaccard coefficient, and the Bray–Curtis dissimilarity index.

### Histopathology

Intestinal injury was graded by a blinded reviewer on a 5-point scale (Supplementary Fig. 2a): grade 0: no injury; grade 1: injury to villus tips or colonic epithelium, or mild separation of lamina propria; grade 2: mid-villus disruption and/or moderate separation of lamina propria; grade 3: complete villus disruption and/or severe separation and/or edema in submucosa; grade 4: transmural injury.

### Immunohistochemistry

Formalin-fixed, paraffin-embedded tissues were deparaffinized and processed for antigen retrieval (EZ-AR Common solution; Biogenex, San Remon, CA), digested with proteinase K (20 µg mL^−1^, 10 min; Promega, Madison, WI), and blocked for 30 min (SuperBlock T20 blocking buffer; Thermo Fisher). The slides were then incubated overnight at 4 °C with primary antibodies: rat anti-mouse F4/80 (dilution 1:50; clone BM8; catalog #14-4801-82; Thermo Fisher), rabbit anti-mouse Ly6C (dilution 1:100; Abcam, Cambridge, MA; catalog #ab77766); goat anti-ZO-1 (dilution 1:100; Abcam; catalog #ab190085), rabbit anti-occludin (dilution 1:100; Abcam; catalog #ab31721), rabbit anti-myeloperoxidase (dilution 1:100; Abcam; catalog #ab45977), and anti-4-HNE (dilution 1:200; Sigma, Burlington, MA; catalog #AB5605). Antibodies were selected after reviewing the validation information provided on the manufacturer’s websites; each clone has been validated by multiple methods. Secondary staining was performed with Alexa 488- or Alexa 568-conjugated antibodies for 30 min (Invitrogen, San Diego, CA). Coverslips were mounted using a liquid mountant containing 4′,6-diamidino-2-phenylindole (DAPI; Prolong Gold Antifade Mountant; Thermo Fisher; catalog #P36930) and the tissues were imaged using a Leica SP2 confocal microscope.

To visualize products of lipid peroxidation in cell membranes, tissues were fixed in 2% paraformaldehyde for 4 h and then embedded in 2% low melting-point agarose (Thermo Fisher; catalog #16520100) dissolved in PBS. The agarose block was sectioned into 40–100 µm slices using a vibratome (Leica Microsystems, Buffalo, NY) and stained in a 24-well plate with anti-4-HNE (dilution 1:100; Sigma; catalog #AB5605), and with either rabbit anti-mouse Ly6C (dilution 1:100; Abcam; catalog #ab77766) or rabbit anti-mouse myeloperoxidase (dilution 1:100; Abcam; catalog #ab45977). The agarose slices were mounted on glass slides using a spacer and a coverslip. Imaging was done using a multiphoton Laser-scanning microscope (Olympus FV1000 MPE, Pittsburgh, PA).

### Reverse transcriptase-quantitative polymerase chain reaction

Total RNA was extracted from cells using RNeasy Mini Kit (Qiagen, Germantown, MD; catalog #74104) according to the manufacturer’s protocol. First-strand complementary DNA was synthesized using oligo-dT primers and Moloney murine leukemia virus reverse transciptase (Superscript II; Thermo Fisher; catalog #18064014). Primers were designed using the Beacon Design software (Bio-Rad, Hercules, CA) and are listed in Supplementary Table 3. PCR amplification was performed on a My-IQ thermocycler (Bio-Rad, Hercules, CA) using the SsoAdvanced Universal SYBR Green supermix (Bio-Rad; catalog #1725271). Amplification efficiency was controlled by the use of an internal control (18S rRNA). Relative quantification of target mRNA expression was calculated and normalized to 18S rRNA expression. The 2–ΔΔCT method was used to compare gene expression levels between samples, which are presented as the fold induction of mRNA expression relative to the amount present in control samples.

### Protein measurements

Enzyme immunoassays were used to measure plasma concentrations of murine fatty acid-binding protein 2 (Lifespan Biosciences, Seattle, WA; catalog #LS-F11419), C-reactive protein, (Lifespan Biosciences; catalog #LS-F4264), serum amyloid A (Lifespan Biosciences; catalog #LS-F20766), and Cxcl2 (R&D Systems, Minneapolis, MN; catalog #MM200).

### Flow cytometry

Intestinal tissue was washed with Hank’s balanced salt solution containing 1 mM dithiothreitol (Sigma) to remove mucus, and then treated first with HBSS containing 1 mM EDTA (Sigma) for 20 min at 37 °C, and then with HBSS containing 1 mM collagenase type IV (Sigma) for 2 h at 37 °C. Cell suspension was stained with the L-D viability dye (Thermo Fisher; catalog #L34957) and antibodies against CD45 (dilution 1:25; clone #30-F11), CD11b (dilution 1:25; clone #M1/70), Ly6C (dilution 1:25; clone #HK1.4), F4/80 (dilution 1:25; clone #BM8), Ly6G (dilution 1:25; clone #1A8; all from Biolegend, San Diego, CA), and Ly6B (dilution 1:25; Bio-Rad, Hercules, CA; catalog #MCA771G). Data were acquired on a BD LSR-II flow cytometer and analyzed using the software package FlowJo version 10.5.3 (Becton Dickinson, Franklin Lakes, NJ).

### Murine intestinal macrophages

Macrophages were isolated from enzymatically digested intestinal tissue by immunomagnetic separation using anti-Ly6C-PE (clone #HK1.4; BioLegend, catalog #128015) and anti-PE ferromagnetic beads (Miltenyi Biotec, San Diego, CA; catalog #130-048-801).

In some experiments, we treated mice with liposome-encapsulated clodronate (ClodronateLiposomes.com Inc., Brentwood, TN; 50 mg kg^−1^ intraperitoneal and 50 mg kg^−1^ by enema on P8). This treatment consistently depleted >80% monocytes and macrophages within 72 h.

### Cell-free hemoglobin and heme

Stored RBCs and associated supernatants, RBCs, and plasma collected from transfused pups were sent on ice to the University of Alabama at Birmingham. Levels of free hemoglobin and heme were measured immediately upon receipt using spectral deconvolution^48^. Spectra were collected between 450 nm and 700 nm for the following preparations: (i) stored RBCs (1 µL of RBC hemolysates diluted into 1 mL PBS; 1 cm cuvette); (ii) stored RBC supernatants (10 µL of samples added to 990 µL PBS; 1 cm cuvette); (iii) plasma (20 µL added to 80 µL PBS; 1 mm cuvette). In each case, spectral deconvolution was performed using the entire spectrum except for mouse plasma where 520–700 nm spectra were used. Deconvolution was performed using Excel with a combination of standard spectra (of deoxyHb, oxyHb, metHb, cyanmetHb, and hemin) that resulted in the best fits determined by least squares fitting analyses.

### Reporter cell lines and siRNA

NF-κB/secreted alkaline phosphatase (SEAP) reporter RAW264.7 cells (Novus Biologicals; catalog #NBP2-26260) are stably ransfected to express the SEAP reporter gene under the transcriptional control of an NF-κB response element. HEK-Blue-mTLR4 cells (Invivogen; catalog code hkb-mtlr4) are co-transfected with murine TLR4, MD-2, CD14, and inducible secreted embryonic alkaline phosphatase reporter gene. Cells were used at passages 1–3 after purchase. Validation data from the manufacturer were reviewed; further authentication (other than inclusion of positive and negative controls) was not performed. siRNA against murine Tlr4, Myd88, and Ticam1 were purchased from Thermo Fisher (catalog #AM16708). Mycoplasma testing was performed by PCR (Agilent Technologies; catalog #302106).

### Nanoparticles with anti-NF-κB p65 siRNA

The p5RHH siRNA NPs were assembled by mixing the p5RHH peptide (VLTTGLPALISWIRRRHRRHC; GenScript, Piscataway, NJ; dissolved at 10 mM in DNAse-, RNAse-, and protease-free sterile purified water, Thermo Fisher; catalog #10977015) in equal volumes with Cy3-labeled anti-NF-κB p65 (or scrambled) siRNA (Sigma-Aldrich; catalog #EMU076311; dissolved at 100 μM in 1× siRNA buffer; Thermo Fisher, catalog #AM8790G) at a peptide/siRNA ratio of 1:20 in HBSS with Ca^2+^ and Mg^2+^ (Gibco, Life Technologies) and incubated on ice for 10 min. This preparation typically results in a nominal NP size of approximately 55 nm after applying a stabilizing albumin coating, zeta potentials varying from +12 to –5.5 mV, and a polydispersity index of 0.12–0.19 depending on size and the presence or absence of an exogenously applied albumin coating. Doses for in vivo administration and the duration of action were based on pilot studies^52^.

### Statistical methods

Statistical analysis was performed using the GraphPad Prism software, version 7.01 (GraphPad, La Jolla, CA, USA). All tests were two sided. Differences were considered significant at *P* < 0.05.

## Supplementary information

Supplementary information

Source data

## Data Availability

Data used in this study have been collected in a preclinical study approved by the Institutional Animal Care and Use Committees at the University of South Florida and Johns Hopkins University. Data are available at the public repository Figshare (https://figshare.com/s/bb85108cf4a4d9455445) and also upon request from the corresponding author. Microbiome data have been uploaded to the NCBI Sequence Read Archive under the BioProject ID PRJNA541440 with the following accession numbers: SAMN11585257, SAMN11585274, SAMN11585275, SAMN11585277, SAMN11585279, SAMN11585280, SAMN11585282, SAMN11585283, SAMN11585284, SAMN11585285, SAMN11585286, SAMN11585289, SAMN11585287, SAMN11585326, SAMN11585331, SAMN11585332, SAMN11585333, SAMN11585345, SAMN11585284, and SAMN11585347.
