# Supplementary material for: A murine neonatal model of necrotizing enterocolitis caused by anemia and red blood cell transfusions
Source: Nat Commun. 2019 Aug 2;10:3494. doi: 10.1038/s41467-019-11199-5 (PMC6677753; doi:10.1038/s41467-019-11199-5)
Supplement: Supplementary file 1 — Supplementary information [file 41467_2019_11199_MOESM1_ESM.pdf]

A Murine Neonatal Model of Necrotizing Enterocolitis Caused by Anemia and Red Blood Cell Transfusions

MohanKumar *et al.*

Supplementary Information

Supplementary Figure 1a. Gut microbiome of P10 mouse pups inoculated with *Serratia marcescens*.

Supplementary Figure 1b. Buffy coat removal from donor mouse blood for leukoreduction.

Supplementary Figure 2a. Histopathological grading for intestinal injury.

Supplementary Figure 2b. Intestinal injury in control and Serratia-colonized mice.

Supplementary Figure 3a. Immunohistochemistry for tight junction proteins.

Supplementary Figure 3b. Intravenously administered polystyrene microspheres lodged in the distal ileum.

Supplementary Figure 3c. Seeding density of polystyrene microspheres in duodenum, jejunum, and colon.

Supplementary Figure 4a. FACS gating strategy for CD11b (+) myeloid cells.

Supplementary Figure 4b. FACS enumeration of macrophage subpopulations in the anemic-transfused intestine.

Supplementary Figure 4c. FACS gating strategy for neutrophil populations in the intestine.

Supplementary Figure 4d. FACS enumeration of neutrophil populations in the anemic-transfused intestine.

Supplementary Figure 4e. Intestinal injury in anemic-transfused RAG1 (-/-).

Supplementary Figure 5a. 4-hydroxynonenal staining in anemic and anemic-transfused intestine.

Supplementary Figure 5b. Neutrophil activation in the anemic-transfused intestine.

Supplementary Figure 5c. Intestinal injury following allogeneic *vs*. syngeneic RBC transfusions.

Supplementary Figure 5d. Plasma haptoglobin concentrations control and anemic-transfused mice.

Supplementary Figure 6a-c. Inflammatory cytokines, TLR-activated, and NF-kappa B pathway genes in transfusion-associated intestinal injury.

Supplementary Figure 6d. FACS enumeration of macrophage subpopulations in the TLR4 (-/-) intestine.

Supplementary Figure 7a. FACS enumeration of intestinal myeloid cells after clodronate treatment.

Supplementary Figure 7b. Macrophage depletion in CSF1R-DTR/mCherry × Lyz2-cre mice.

Supplementary Figure 7c. FACS enumeration of macrophages in CSF1R-DTR/mCherry × Lyz2-cre mice.

Supplementary table 1. Hematocrit and red cell indices in controls and anemic-transfused pups

Supplementary table 2. Serial weights (mean ± SE) of mouse pups in the 4 experimental groups

Supplementary table 3. Primers used for quantitative PCR

Supplementary Figure 1a.

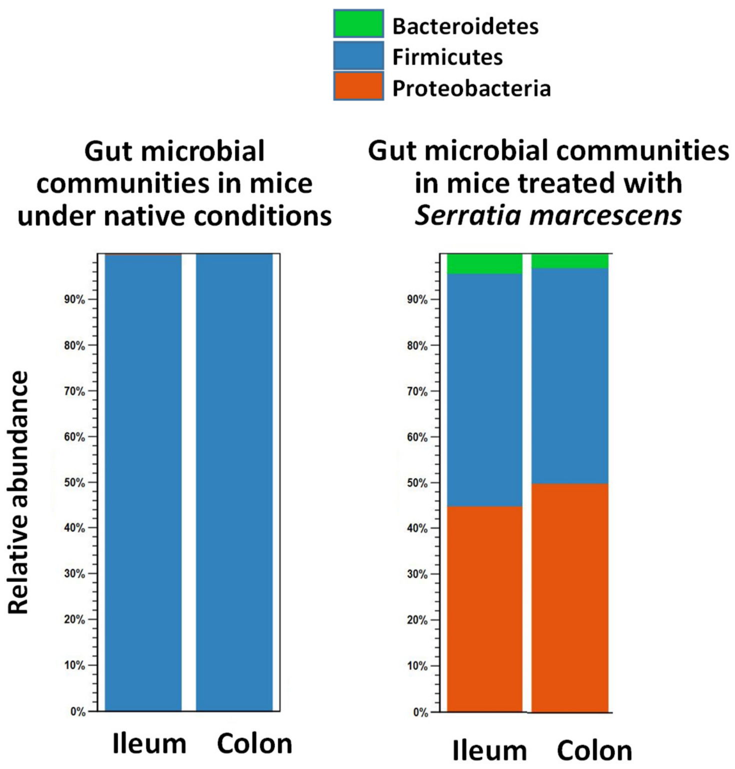

Bacterial taxonomic profiles in the intestine from mice reared under conventional conditions and in those given *Serratia marcescens* (10^4 colony forming units by gavage) on P7. Tissue samples were harvested on P10 and analyzed by PCR amplification of the 16S rRNA gene. In mice inoculated with *Serratia,* the predominance of these bacteria in the Proteobacteria fraction on P10 was confirmed by analysis at genus level. *N*=10 mice/group.

Supplementary Figure 1b.

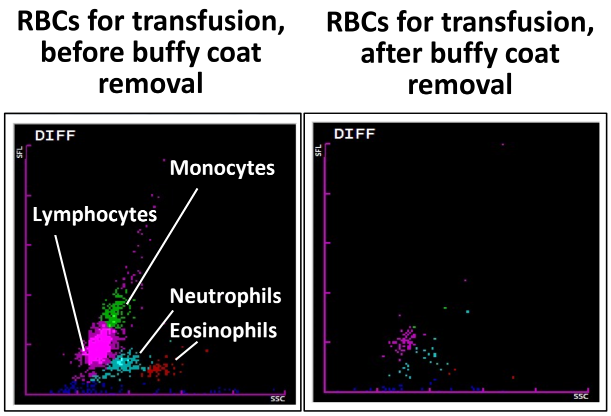

Representative differential scattergrams of RBCs being prepared for transfusions show that the removal of the buffy coat (along with some plasma and top part of the RBC layer) removed >99% of the leukocytes.

Supplementary Figure 2a.

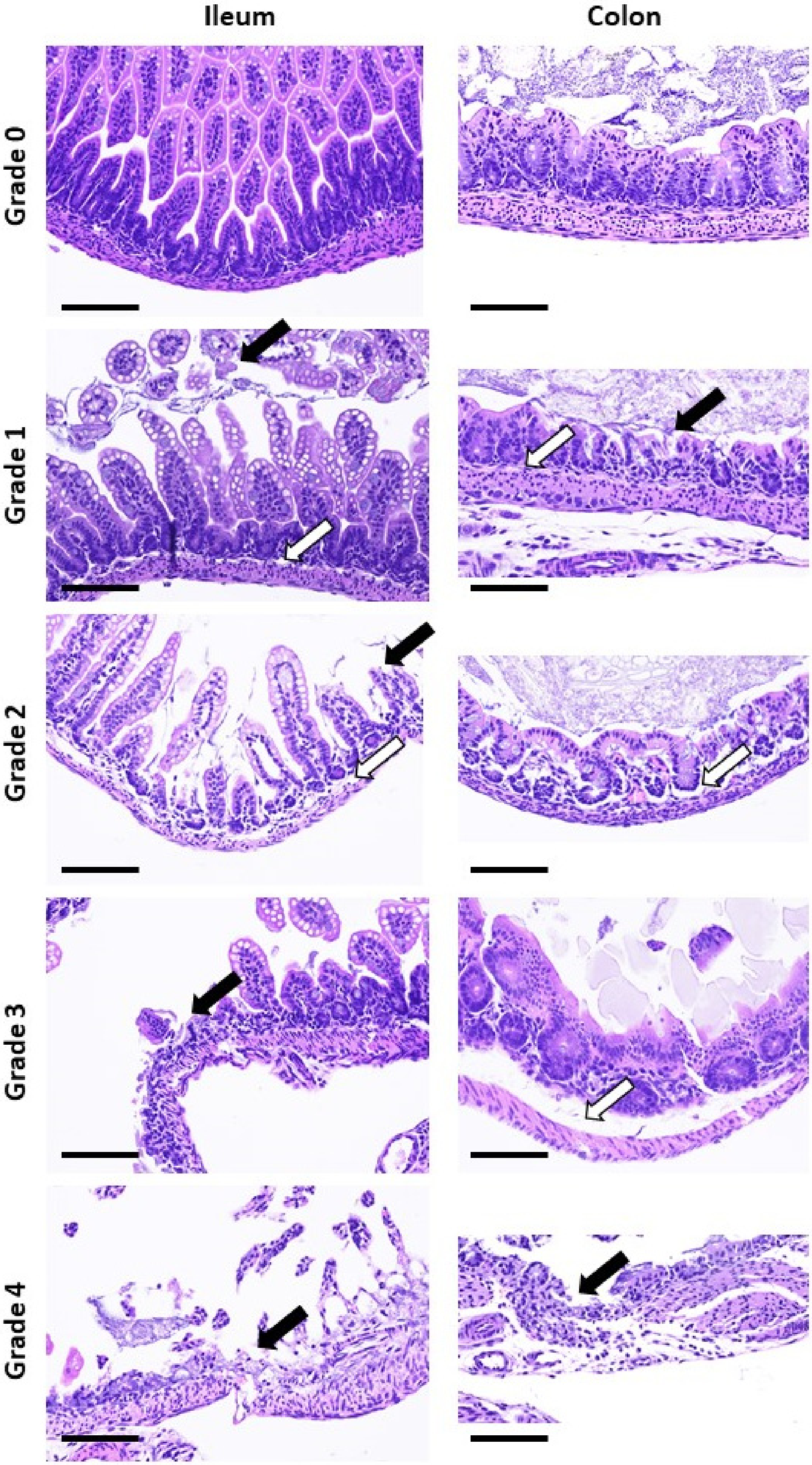

Grading of RBC transfusion-associated intestinal injury: grade 0: no injury; grade 1: injury to villus tips or colonic epithelium, or mild separation of *lamina propria*; grade 2: mid-villus disruption and/or moderate separation of *lamina propria* in the small intestine or colon; grade 3: complete villus disruption and/or severe separation and/or edema in submucosa; grade 4: transmural injury. H&E-stained photomicrographs (magnification 200x) of ileum (*left*) and colon (*right*) from anemic-transfused mouse pups show various injury grades. Grade 0 shows intact ileum and colon. Grade 1 injury was localized to villus tips (solid black arrow) and mild separation of the *lamina propria* (white arrow). Colon shows focal epithelial injury (black arrow) and minimal separation of the *lamina propria*. Images with grade 2 injury show moderate separation of the *lamina propria* (white arrow) and mid-villus injury (black arrow). Colon shows moderate separation of the *lamina propria* (white arrow) and more extensive epithelial damage than grade 1. Grade 3 shows complete disruption of the crypt-villus axis (black arrow) and severe separation of the *lamina propria* (white arrow) in the colon. Inflammatory changes are prominent. Grade 4 shows transmural necrosis in both ileum and colon (black arrows). Scale bar = 200 µm. N = 10 mice.

Supplementary Figure 2b.

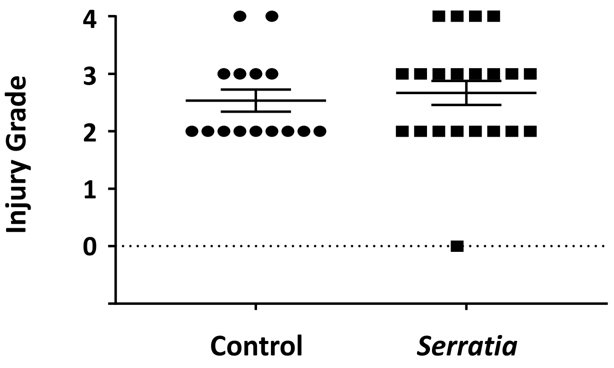

Scatter plots (means ± SE) summarize the severity of bowel injury in mice colonized with Serratia *vs*. control mice that were not exposed to these bacteria. *N* = 15 controls, 21 colonized with Serratia. Mann-Whitney *U* test; not significant.

Supplementary Figure 3a.

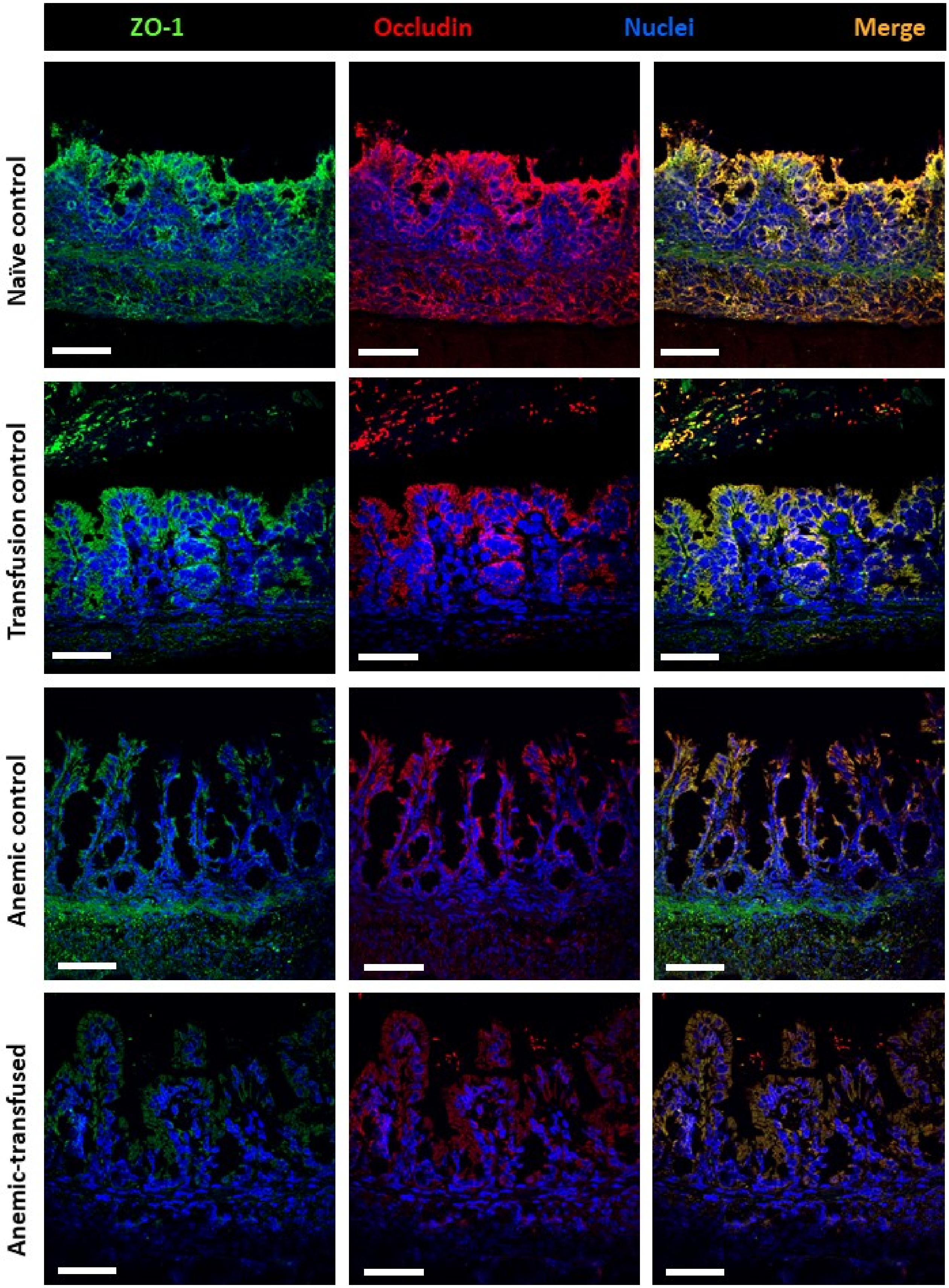

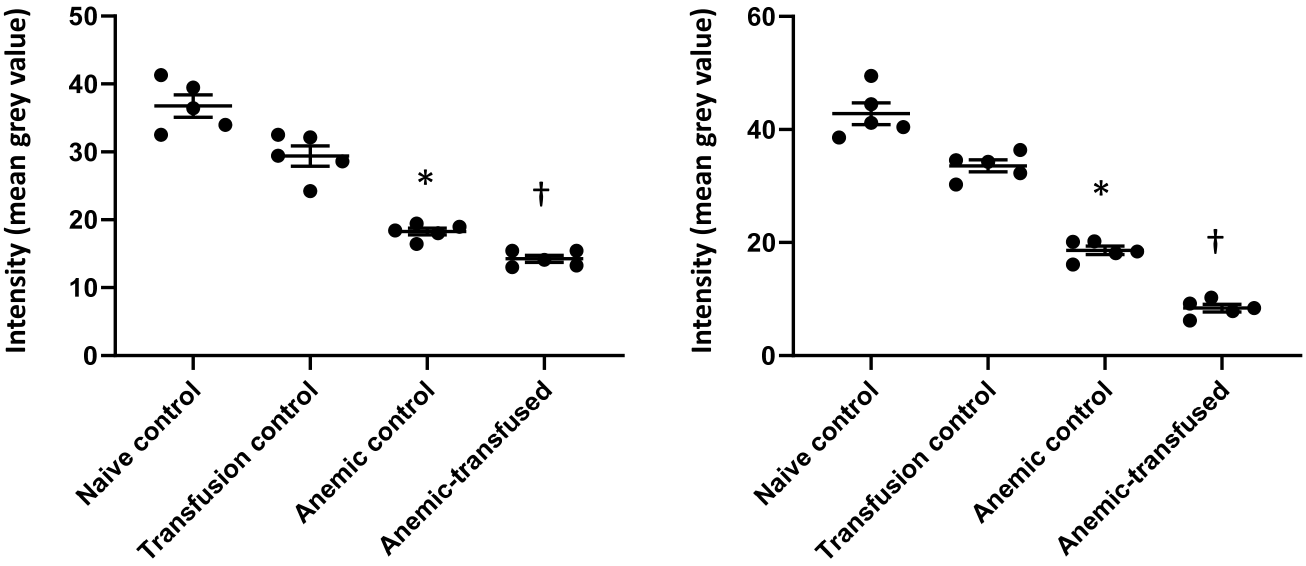

Fluorescence photomicrographs (magnification 250x) of proximal colon (*bottom*) show immunoreactivity for the tight junction proteins ZO-1 (zonula occludens-1; green), occludin (red), and a computer-assisted merge in naïve control, transfusion control, anemic control, and the anemic-transfused mice. Nuclear staining (blue) was obtained with DAPI. Scatter plots below summarize the fluorescence intensity data for ZO-1 and occludin in the 4 groups. Scale bars = 50 µm. Data represent 5 mice/group. Kruskal-Wallis *H* test with Dunn's post-test, ** P<0.01, † P<0.001 *vs*. naïve control.

Supplementary Figure 3b.

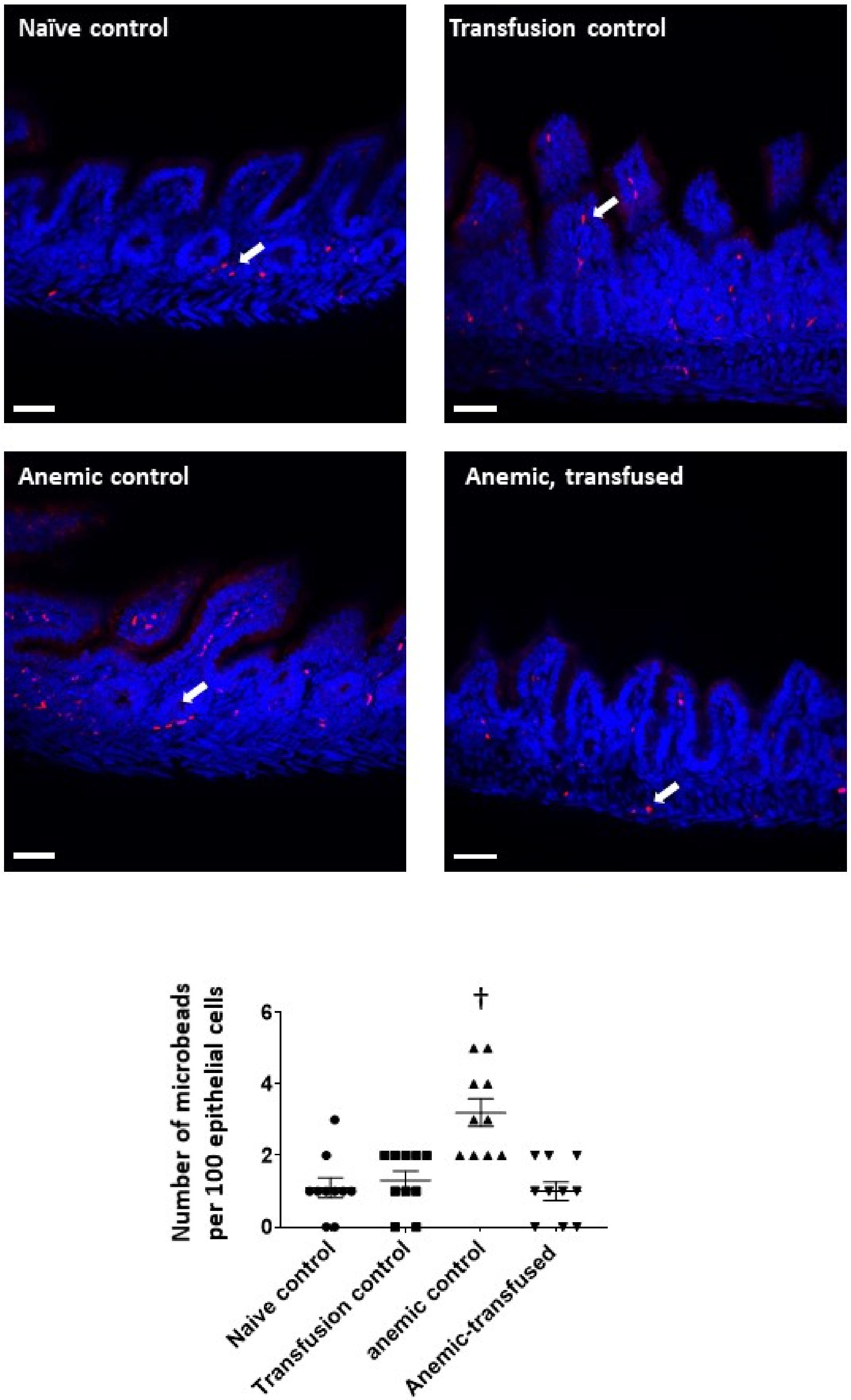

Fluorescence photomicrographs (250x) from naïve control, transfusion control, anemic control, and the anemic-transfused mice show fluorescence-labeled polystyrene microspheres (red) lodged in the distal ileum. Nuclear staining (blue) was obtained with DAPI. Data represent stained sections from 5 mice/group. Bottom: Scatter plots (means ± SE) summarize the number of microspheres per high power field. Kruskal-Wallis *H* test with Dunn's post-test, † (dagger) $P<0.001$ *vs*. naïve control.

Supplementary Figure 3c.

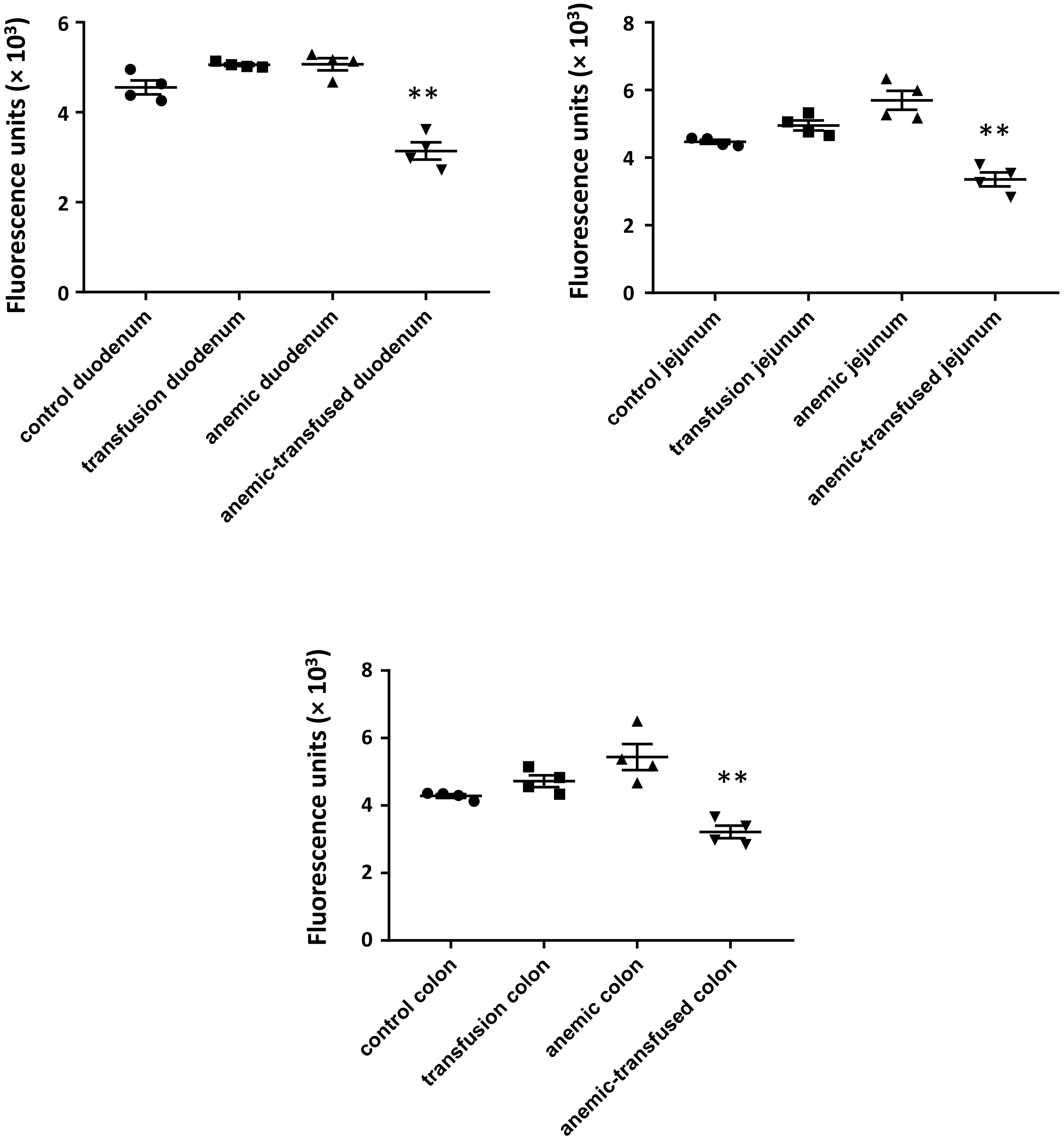

Seeding density of fluorescence-labeled polystyrene microspheres in duodenum, jejunum, and colon from naïve control, transfusion control, anemic control, and the anemic-transfused mice. Scatter column plots summarize data from 8 mice/group. Kruskal-Wallis *H* test, ** $P<0.01$ *vs*. anemic mice.

Supplementary Figure 4a.

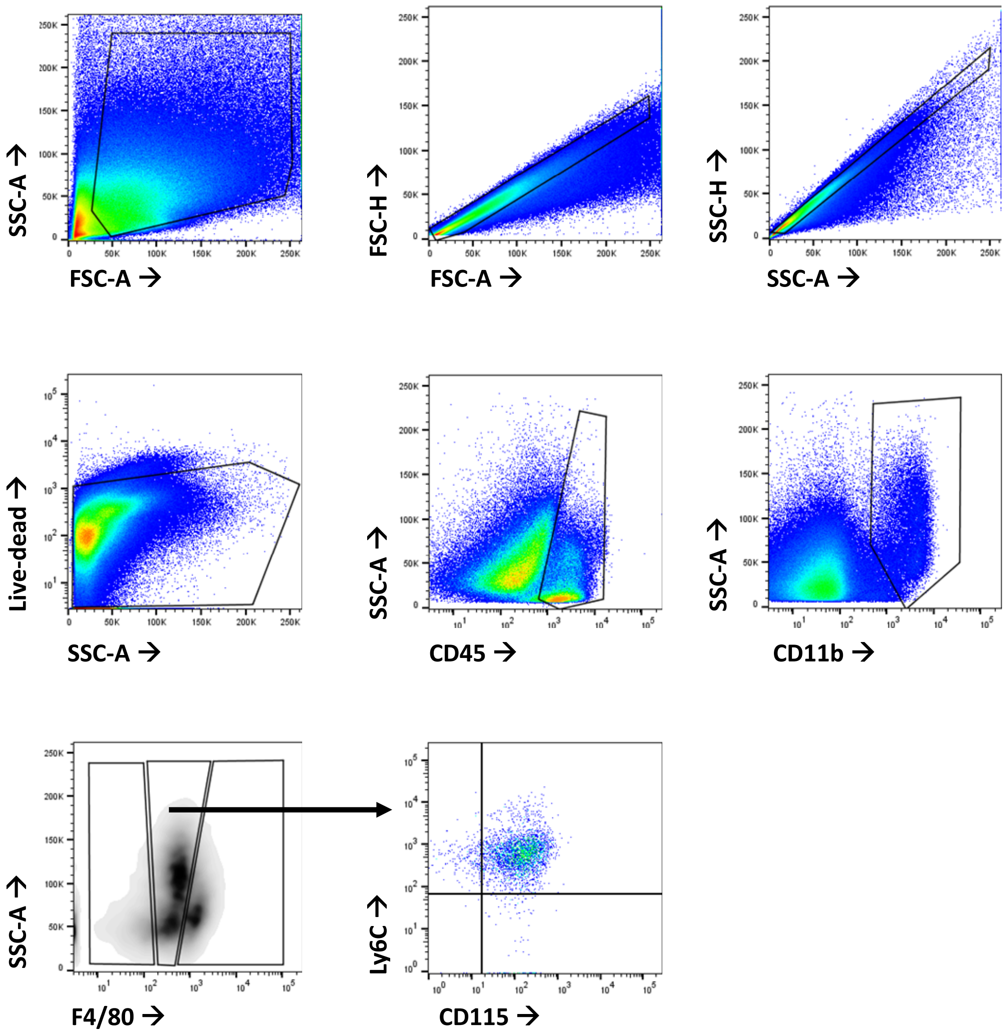

FACS gating strategy used to analyze the CD11b (+) myeloid cell fraction in intestinal cells. Cells were gated based on size and granularity using FSC-A vs SSC-A to eliminate debris and clumped cells. Cell clumps were further excluded in FSC-A *vs*. FSC-H and SSC-A *vs*. SSC-H gates. Single cells were sub-gated using the fixable live-dead viability dye, and then based on the pan-hematopoietic marker CD45, myeloid cell marker CD11b, and the macrophage marker F4/80. The F4/80 (hi) and F4/80 (mid) cell populations were further evaluated using Ly6C and CD115.

Supplementary Figure 4b.

Scatter plots (means ± SE) summarize flow cytometry data and statistical analysis (clockwise) of CD11b (+),

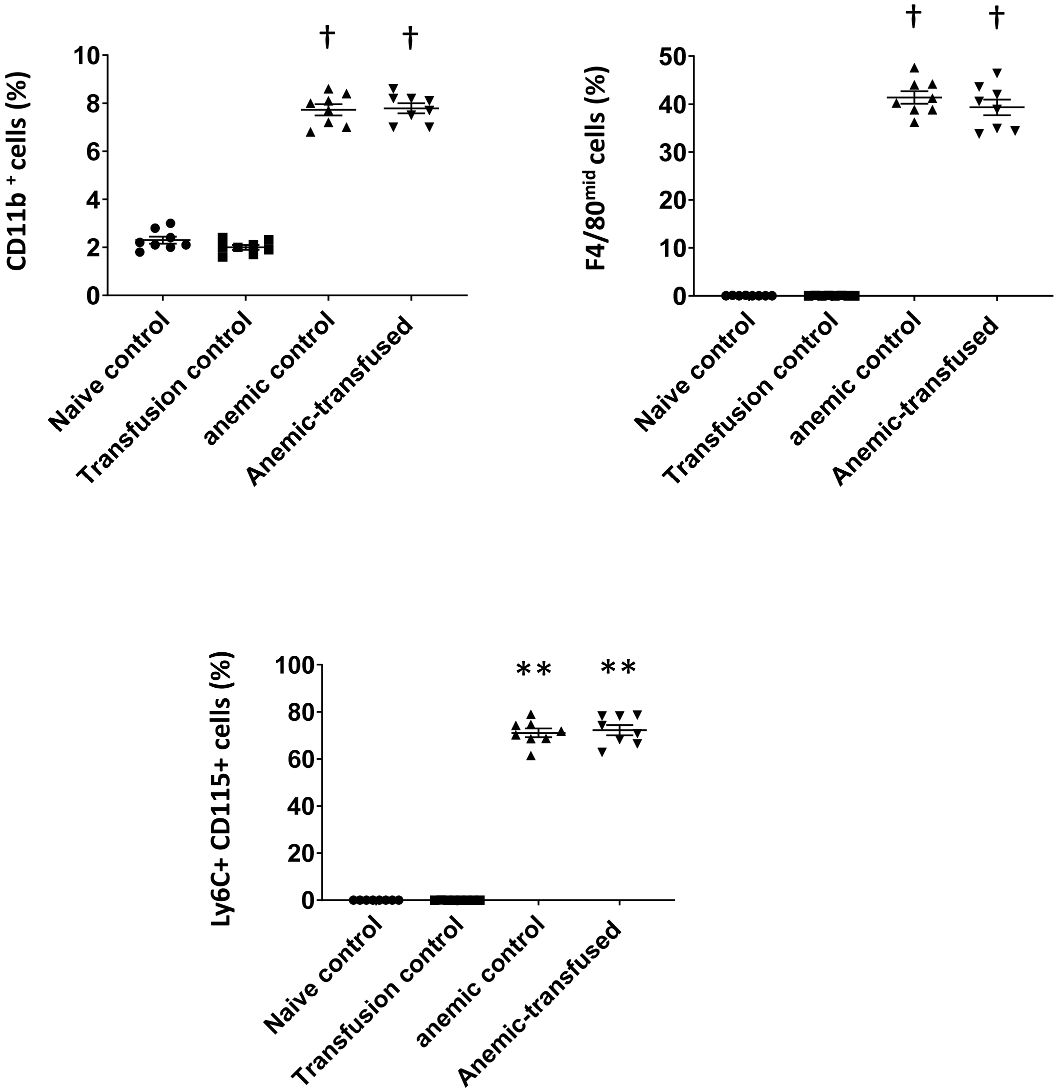

F4/80 (mid), and the Ly6C (+) CD115 (+) cell populations in naïve control, transfusion control, anemic control, and the anemic-transfused intestine, from Figure 4a. Kruskal-Wallis *H* test with Dunn's post-test, ** *P*<0.01, † *P*<0.001 *vs*. naïve control.

Supplementary Figure 4c.

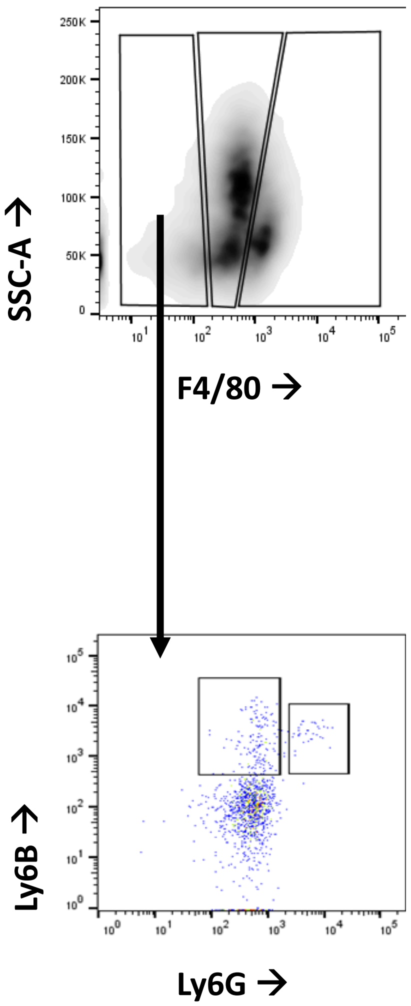

FACS gating strategy used to analyze the neutrophil populations in the intestine. After excluding debris, clumps, and dead cells, single cells were sub-gated based on the pan-hematopoietic marker CD45, myeloid cell marker CD11b, and the macrophage marker F4/80 as shown in Supplementary figure 4a. The F4/80 (low) cell population was further evaluated using Ly6G and Ly6B.

Supplementary Figure 4d.

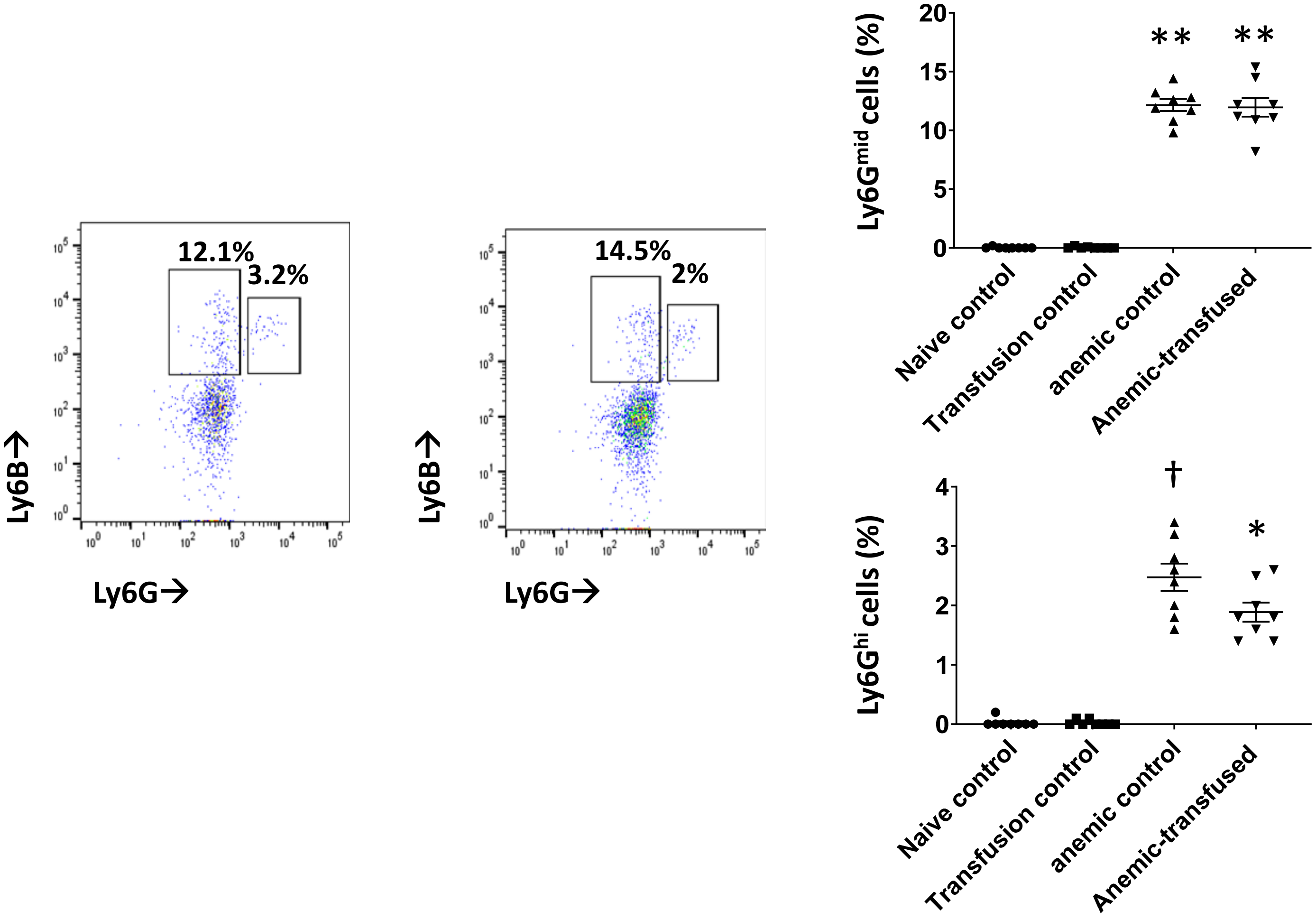

Flow cytometry scatter plots and statistical analysis of F4/80 (low) cells, which included Ly6G (mid) Ly6B (+) late-lineage neutrophil precursors (box on left) and a few mature Ly6G (hi) Ly6B (+) neutrophils (box on right); Kruskal-Wallis *H* test with Dunn's post-test, ** $P<0.01$, † $P<0.001$ *vs*. naïve control. $N = 8$ mice/group.

Supplementary Figure 4e.

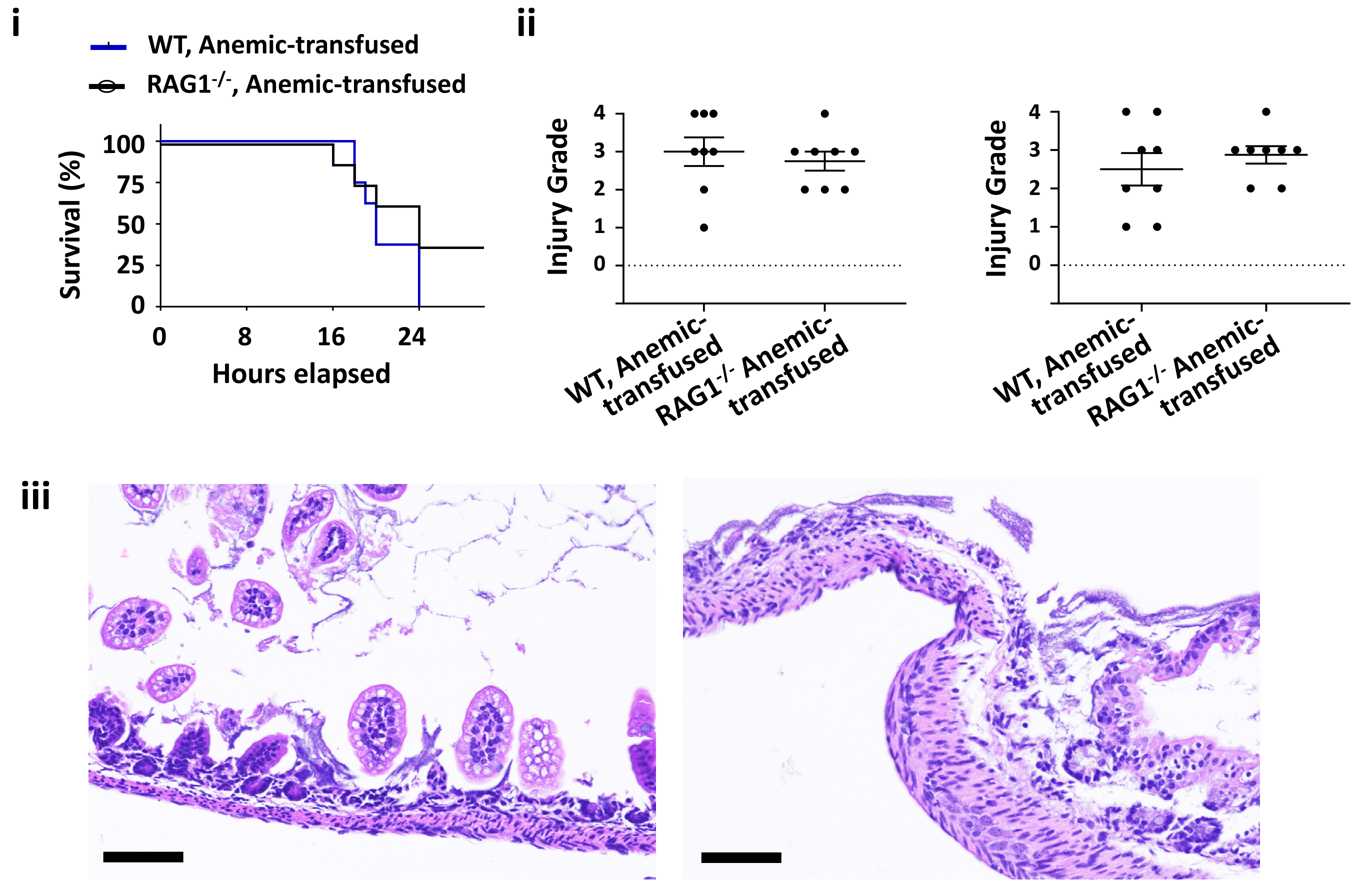

(i) Kaplan-Meier curves show survival without intestinal injury in wild-type (WT) and RAG1(-/-) anemic-transfused mice; *N* = 8 mice, Mantel-Cox log-rank test, not significant; (ii) Scatter plots (means ± SE) show severity of bowel injury in the ileum (left) and colon (right) in wild-type (WT) and RAG1$^{-/-}$ anemic-transfused mice; *N* = 8 mice, Mann-Whitney *U* test, not significant. (iii) Representative photomicrographs (hematoxylin-eosin; magnification 200x) of ileum (*left*) and colon (*right*) show NEC-like injury in RAG1$^{-/-}$ anemic-transfused mice. Scale bars = 200 µm.

Supplementary Figure 5a.

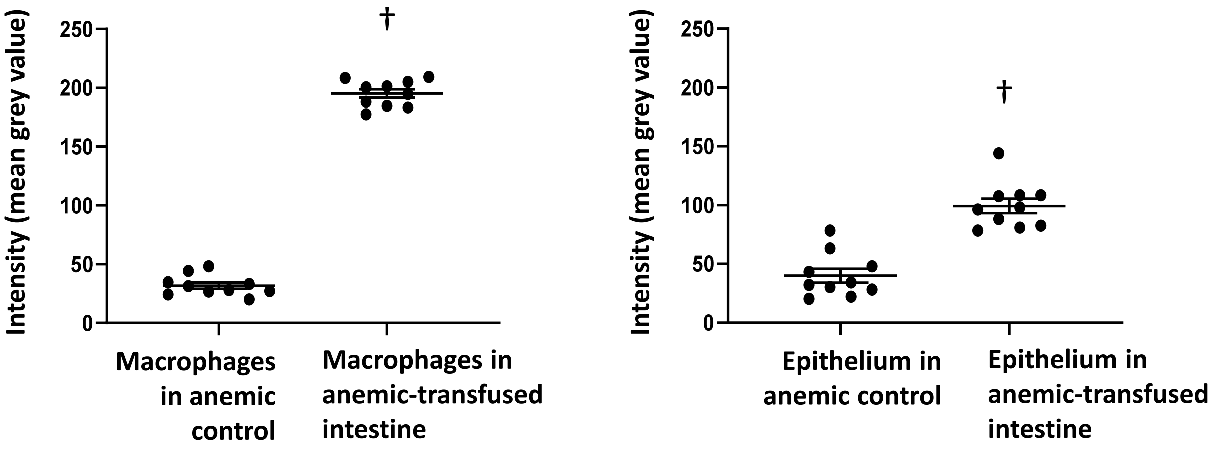

Scatter plots (means ± SE) show fluorescence intensity (mean grey value) for 4-HNE staining of macrophages (left) and the epithelium (right) in the anemic *vs*. anemic-transfused intestine. Mann-Whitney *U* test, † $P<0.001$. $N = 5$ mice/group.

Supplementary Figure 5b.

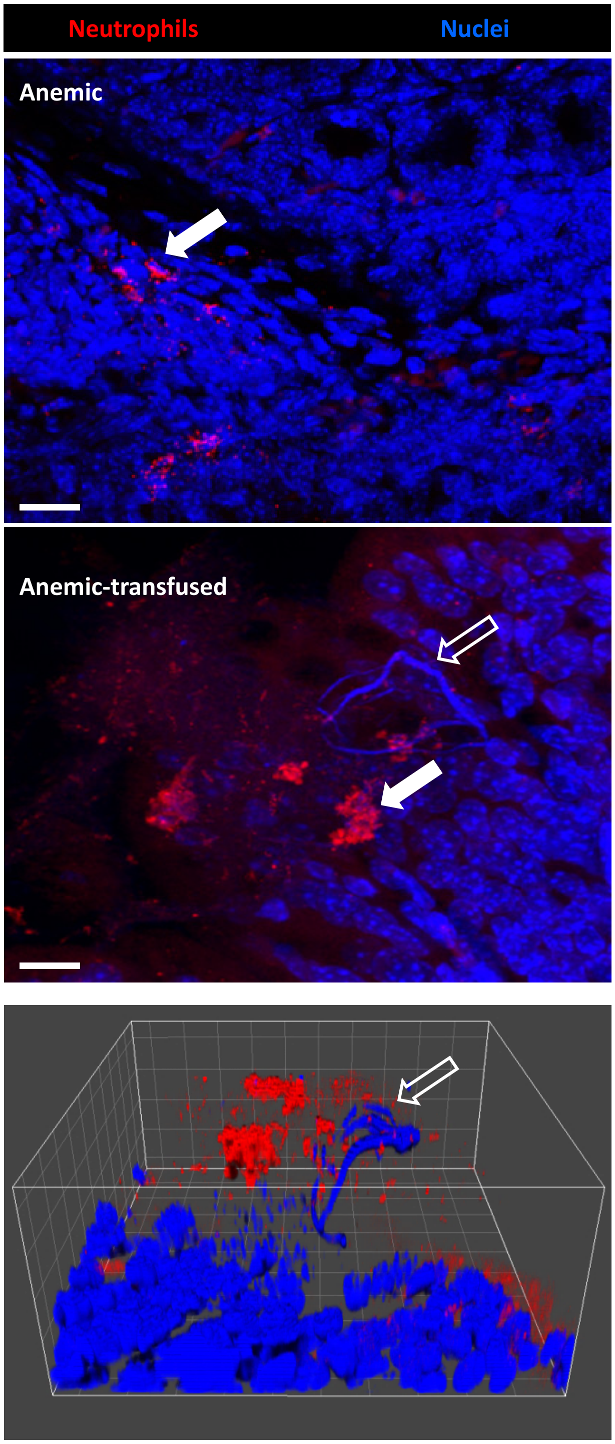

(*Top*) Immunofluorescence photomicrographs (magnification 400x) from the anemic intestine show neutrophils with myeloperoxidase (MPO) staining in discrete cytoplasmic granules. In the anemic-transfused intestine, neutrophils showed an activated appearance with cytoskeletal processes and diffuse MPO staining (solid arrow). Some neutrophils showed extracellular trap formation (open arrow); (*Bottom*) 3D image reconstruction traces the neutrophil extracellular trap (open arrow). Scale bars = 50 µm. Data represent 8 mice per group

Supplementary Figure 5c.

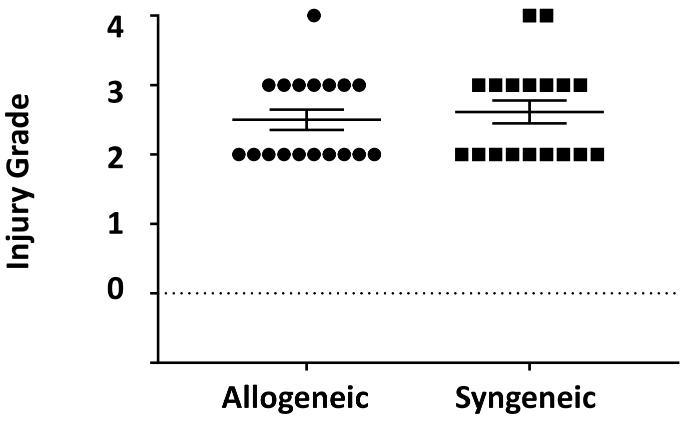

Scatter plots (means ± standard error) show the severity of NEC-like injury following allogeneic (FVB/NJ donors) *vs*. syngeneic (C57BL/6 donors) RBC transfusions. $N = 18$ mice/group. Mann-Whitney U test, not significant.

Supplementary Figure 5d.

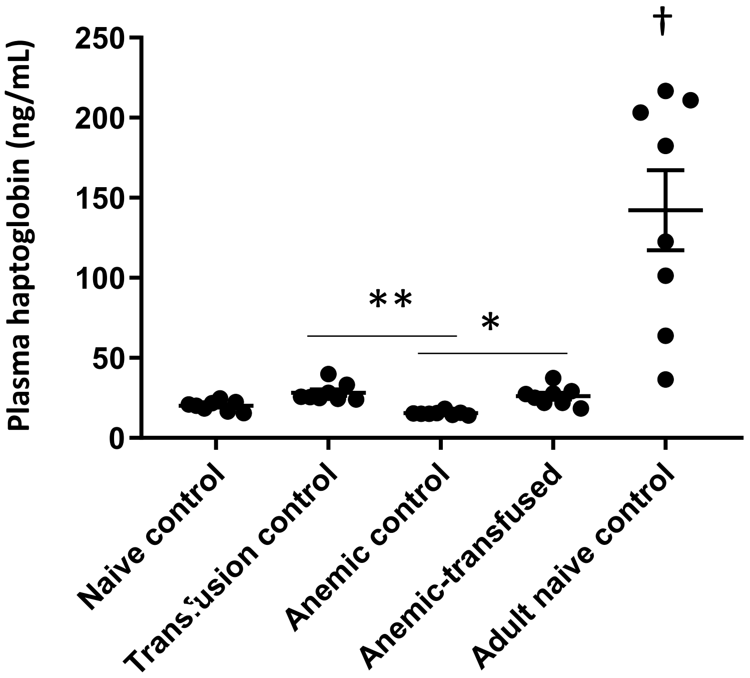

Plasma haptoglobin concentrations in naïve control, transfusion control, anemic control, and anemic-transfused pups. A comparison group of adult mice was also included. Scatter column plots (means ± standard error) summarize data from 8 mice/group. Kruskal-Wallis *H* test with Dunn's post-test, † $P<0.001$ *vs*. naïve control; * $P<0.05$, ** $P<0.01$ between indicated groups

Supplementary Figure 6a.

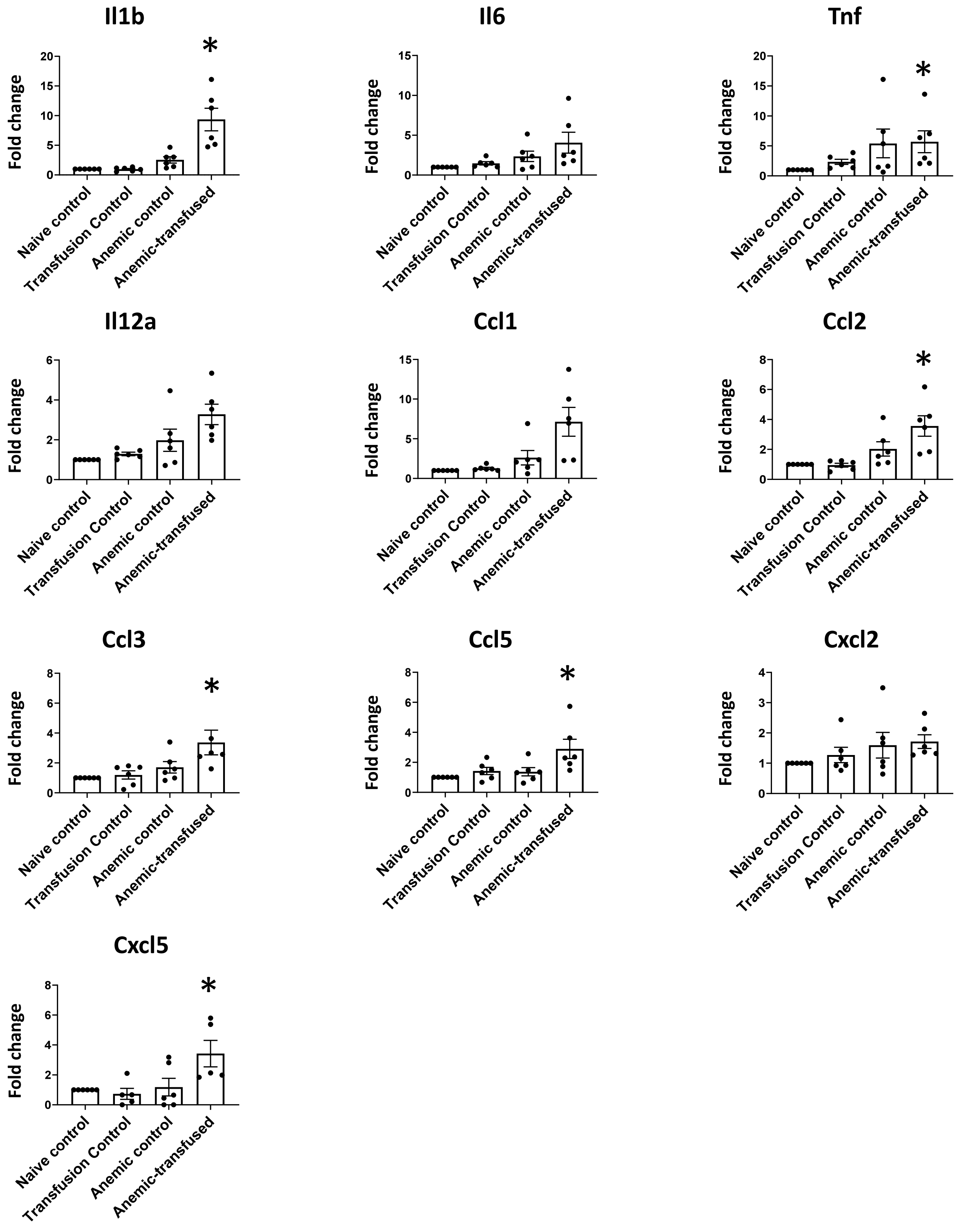

RBC transfusion-associated NEC-like injury induced inflammatory cytokines IL1b, Tnf, Ccl2, Ccl3, Ccl5, and Cxcl5 in the affected intestine. Scatter bar diagrams (means ± standard error of mean) show fold change in mRNA expression above naïve control in transfusion control, anemic control, and anemic-transfused mice. *N* = 8 mice/group, Kruskal-Wallis *H* test, * *P*<0.05 *vs*. naïve control

Supplementary Figure 6b.

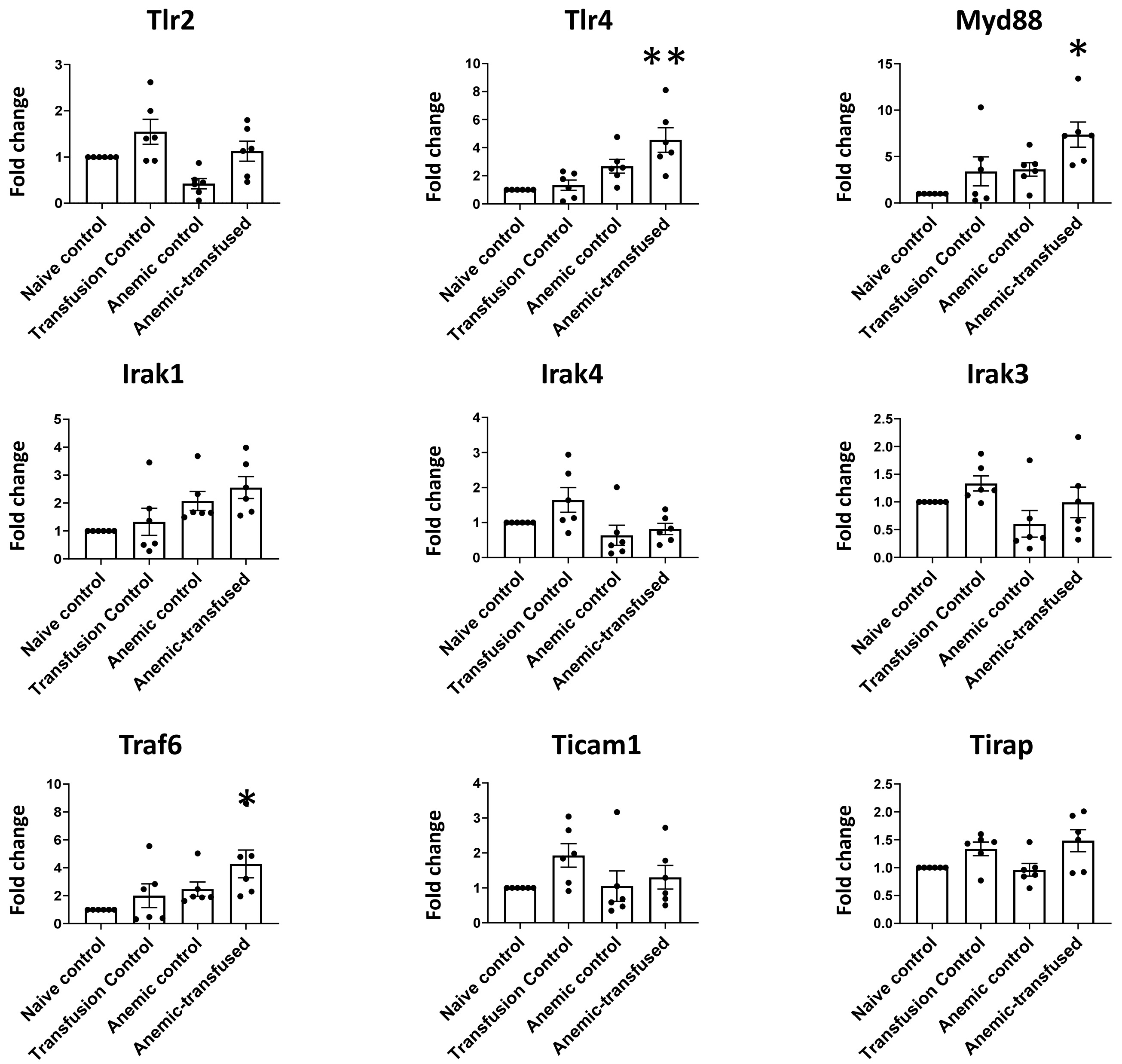

RBC transfusion-associated NEC-like injury induced Toll-like receptor pathway genes Tlr4, Myd88, and Traf6 in the affected intestine. Scatter bar diagrams (means ± standard error of mean) show fold change in mRNA expression above naïve control in transfusion control, anemic control, and anemic-transfused mice. *N* = 8 mice/group, Kruskal-Wallis *H* test, * *P*<0.05 *vs*. naïve control.

Supplementary Figure 6c.

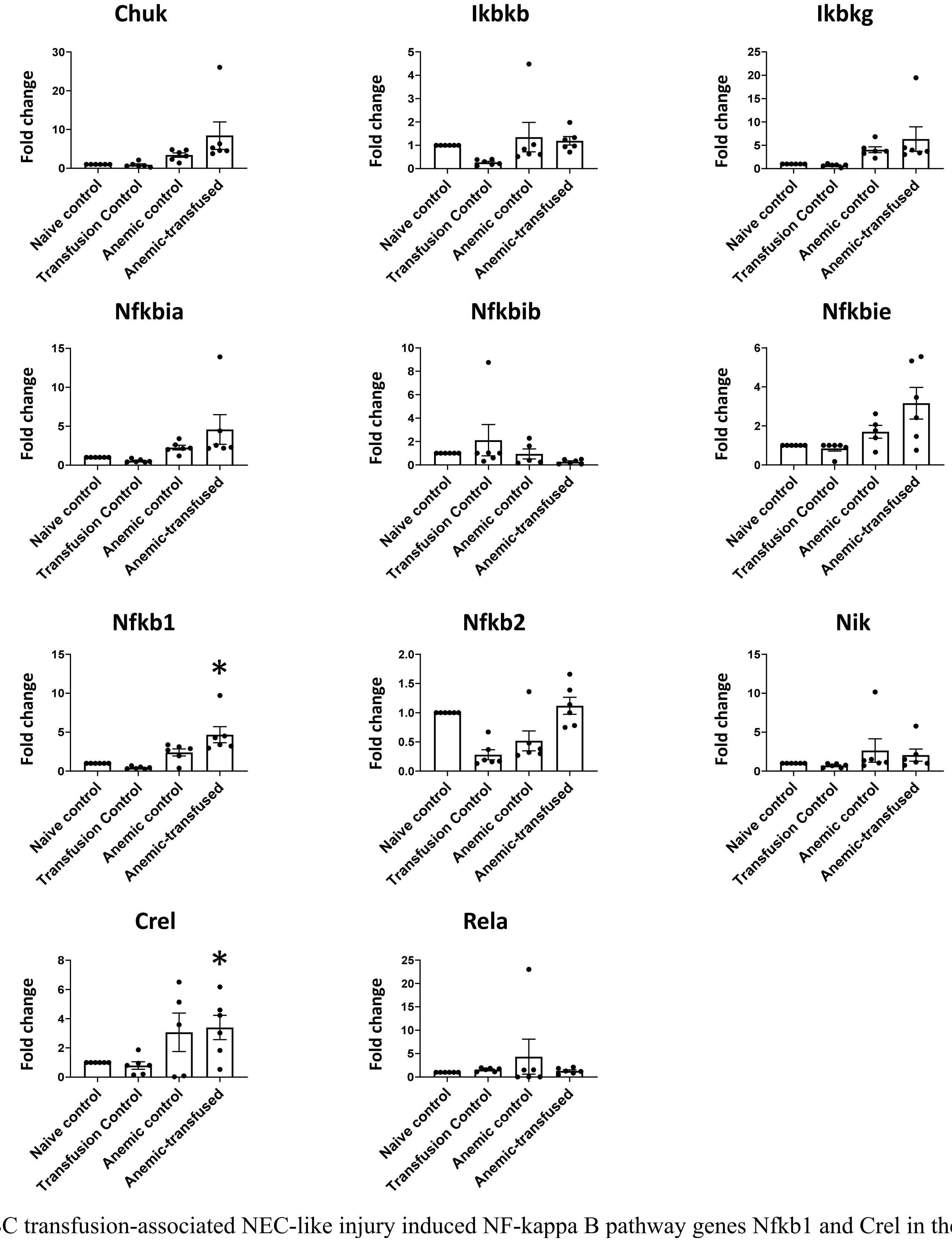

RBC transfusion-associated NEC-like injury induced NF-kappa B pathway genes Nfkb1 and Crel in the affected intestine. Scatter bar diagrams (means ± standard error of mean) show fold change in mRNA expression above naïve control in transfusion control, anemic control, and anemic-transfused mice. *N* = 8 mice/group, Kruskal-Wallis *H* test, * $P<0.05$ *vs*. naïve control.

Supplementary Figure 6d.

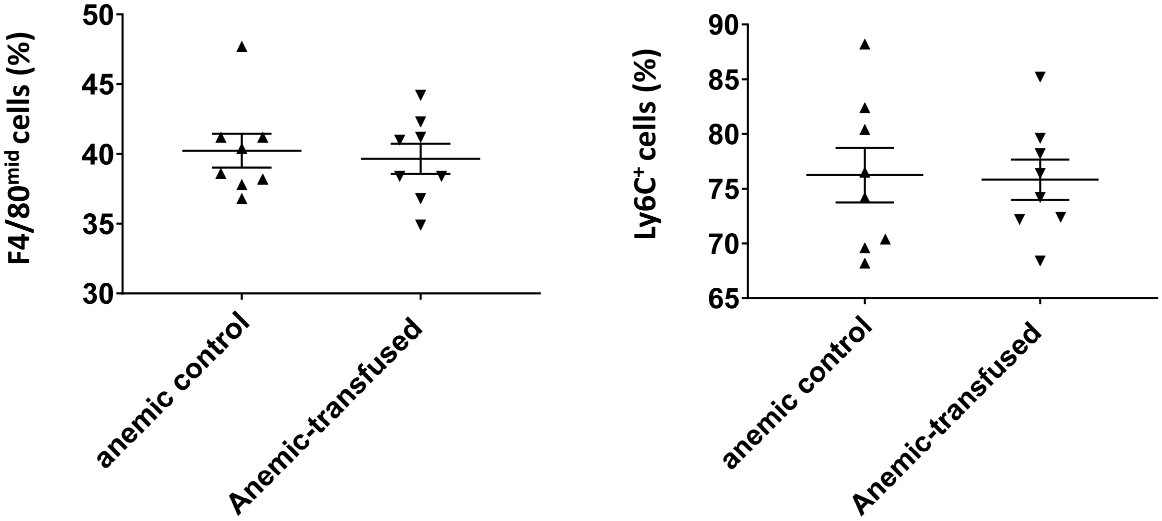

Scatter plots (means ± standard error) summarize flow cytometry data and statistical analysis (clockwise) of F4/80 (mid) and Ly6C (+) CD115 (+) cell populations in TLR4 (-/-) anemic controls and anemic-transfused intestine, depicted in Figure 6g. *N* = 8 mice/group. Mann-Whitney *U* test, not significant.

Supplementary Figure 7a.

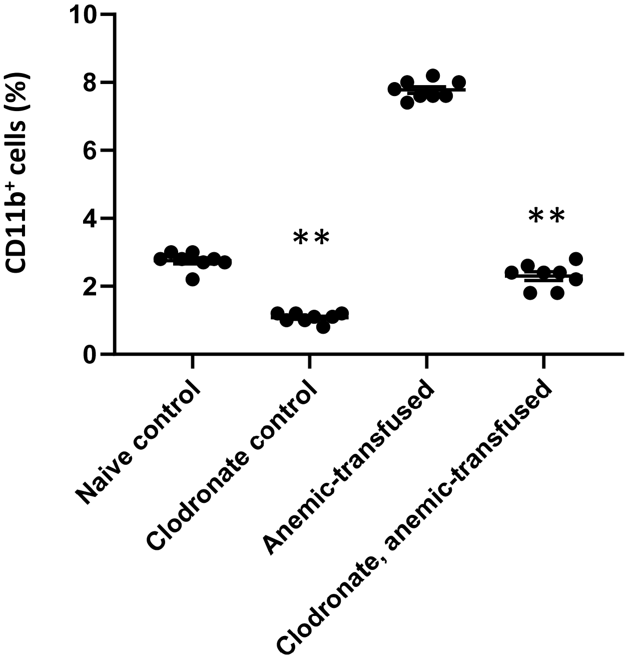

Scatter plots (means ± standard error) summarize flow cytometry data and statistical analysis of CD11b (+) cells in naïve control, clodronate control, anemic-transfused, and clodronate-treated anemic-transfused mice. $N = 8$ mice/group; Kruskal-Wallis H test with Dunn's post-test, ** $P<0.01$.

Supplementary Figure 7b.

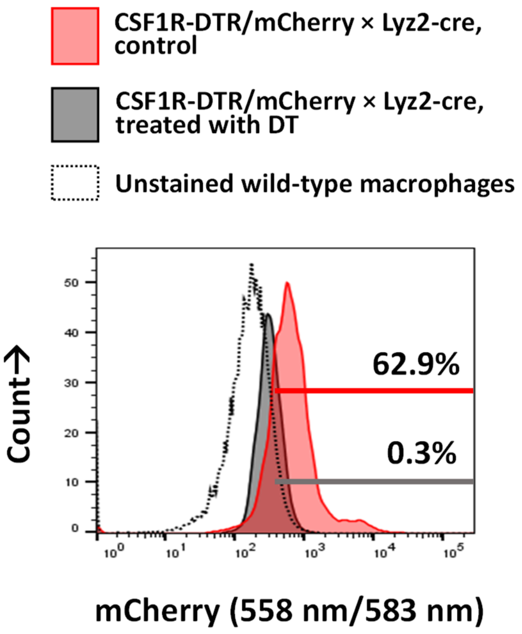

Diphtheria toxin (DT; 5 ng/g body weight, intravenous) depletes all mCherry$^+$ macrophages in CSF1R-DTR/mCherry × Lyz2-cre mice within 24h. Representative histograms (cells gated on CD11b) show the presence of mCherry$^+$ cells in splenic cell suspensions from untreated CSF1R-DTR/mCherry × Lyz2-cre mice (red), but not in mice treated 24h earlier with DT (grey). Unstained wild-type macrophages were used as control (broken outline). $N = 8$ mice/group.

Supplementary Figure 7c.

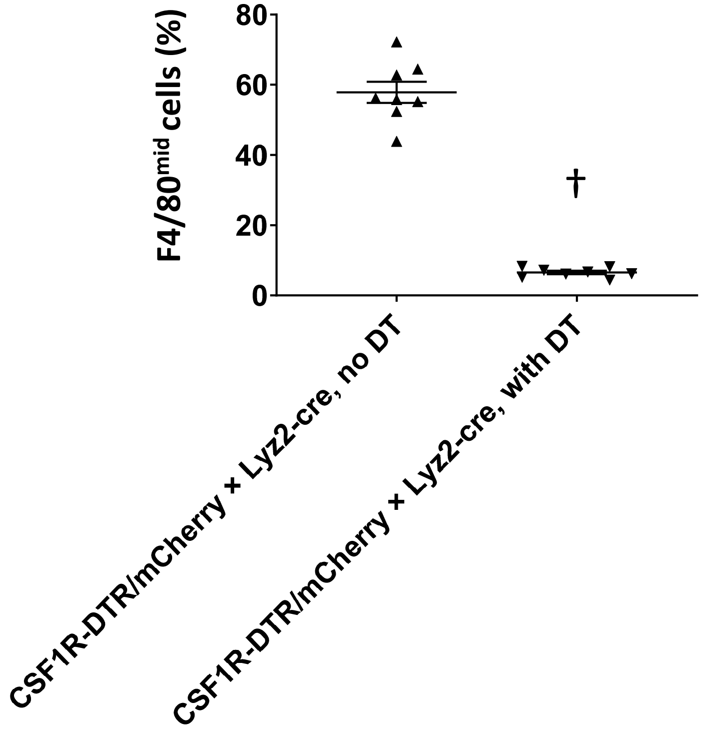

Scatter plots (means ± SE) summarize flow cytometry data and statistical analysis of F4/80^{mid} (Ly6C^{+} CD115^{+}) cell populations in untreated CSF1R-DTR/mCherry × Lyz2-cre mice *vs*. mice treated 24h earlier with DT. $N = 8$ mice/group, Mann-Whitney *U* test, † $P<0.001$.

Supplementary table 1. Hematocrit and red cell indices in controls and anemic-transfused pups

Parameter [median (range)]	Naïve control (*N*=42)	Transfusion control (*N*=21)	Anemic control (*N*=40)	Anemic, transfused (*N*=21)
Hematocrit (%)	45 (42-57)	45 (33-57)	22.5 (18-24)†	27 (18-51)†
Mean corpuscular volume (fL)	51.9 (45.1-75.3)	53.5 (45.9-57.8)	51.8 (42.5-62.5)	50 (44-58.8)
Mean corpuscular hemoglobin (pg)	15.8 (14.3-16.8)	16 (13.8-16.8)	14 (11-15.7)†	14.1 (13.2-15)†
Mean corpuscular hemoglobin concentration (g/dL)	30.6 (26-32.8)	30 (27.1-33.3)	27.3 (24.2-33.3)**	28.4 (23.3-31.2)
Red cell distribution width (fL)	35.6 (26.9-48.5)	32.9 (28.6-37.1)†	38 (32.5-49.3)	33.2 (26.4-52.9)
Reticulocyte count (%)	1.98 (0-2.58)	1.63 (1.2-2.24)	3.37 (0-5.7)†	2.36 (0-5.03)*
Reticulocyte hemoglobin (pg)	13.9 (12.4-16.8)	14 (12.3-15.4)	11.7 (10.7-17)†	12.3 (11.4-19.1)

Kruskal-Wallis H test with Dunn's post-test, * $P<0.05$, ** $P<0.01$, † $P<0.001$.

Supplementary table 2. Serial weights (mean ± SE) of mouse pups in the 4 experimental groups

	P1	P3	P5	P7	P9	P10	P11	P12	P13
Naïve control	1.1±0	1.7±0	2.6±0	3.5±0	4.4±0.1	5.2±0.1	5.6±0.1	6.4±0.1	7.3±0.1
Transfusion control	1.1±0	1.7±0	2.6±0.1	3.6±0.1	4.5±0.1	5.3±0.1	5.7±0.1	6.6±0.1	7.5±0.2
Anemic control	1.1±0	1.6±0	2.4±0.1	3.1±0	4±0.1	4.6±0.1	5±0.1	6.1±0.1	6.1±0.1
Anemic-transfused	1.2±0	1.6±0.1	2.4±0.1	3.2±0	4±0.1	4.8±0.1	5.3±0.1	5.9±0.1	6 ±0.1

Supplementary table 3. Primers used for quantitative PCR

Primer	*Forward*	*Reverse*
Il1b	AATCTATACCTGTCCTGTGTAATG	GCTTGTGCTCTGCTTGTG
Il6	ACAGAAGGAGTGGCTAAGG	GAGAACAACATAAGTCAGATACC
Tnf	AGGACTCAAATGGGCTTTC	AGGTCTGAAGGTAGGAAGG
Il12a	CTCTCATATTCACTATACAAGTTG	GCTCTTCTGCTAACACAT
Ccl1	AGGCTGAACAAAGGTAGAGAAAG	GGTGCTGGGATGGGAAGG
Ccl2	TCCACAACCACCTCAAGCACTTC	GGCATCACAGTTCCGAGTCACACC
Ccl3	GCCTGCTGCTTCTCCTAC	GCTGCCTCCAAGACTCTC
Ccl5	GGTTCAAGAATACATCAACTATT	TAGAGCAAGCAATGACAG
Cxcl2	TCAACGGAAGAACCAAAGAGAAAG	GACAGCGAGGCACATCAGG
Cxcl5	TGTCTTCGTAGTATGGCATAATGT	GCTTCTTACCTTCTTCACCTATCT
Itgam	CTTCCATCCTTCAACACTCAG	TTAGACCTCACATACGACTCC
Tlr4	TTCACCTCTGCCTTCACTAC	GACACTACCACAATAACCTTCC
Myd88	GGAAGAGTTCTATCATCAAGG	GTGTTGCCAAGGATAAGC
Tlr2	ATGCTTCGTTGTTCCCTGTGTTG	AGTGGTTGTCGCCTGCTTCC
Irak1	ATTCCTGGCACTTGACTC	CACACTTGATTCTCTGGTAG
Irak4	TTCCACAGCAACATCACC	GCAGAGACAGGCAGATTG
Irak3	CTTGGTATAGGTGAACAGAGAAC	GACTACTGGCTGCTGATGG
Traf6	TAGTATCCGCATTGAGAAGCC	CCATCCGTGTTAGCAGTTAGG
Trif	TTGCTATGTAGACGAGATTGG	GATTTAGAGAAGGGAGGATTAGG
Tirap	TGGCTGTCTTCTTCCTCTG	AACTTCTGGCTCATCAATCG
Chuk	TGATTGGAGTTGGTTAGCAGAATG	CGGACTGGTAAGTGAGGAATGG
Ikbkb	TGCTGCTTCTCACTTGTCTC	GCCTGTGGAATGTGGATGG
Ikbkg	GGAAGAGCAAGAACAACAAC	CAAGCAACACCAAGGACTACC
Nfkbia	AAGAAGGCGACACAGACC	TTGATTGGAACCACCACCATAGACC
Nfkbib	GGCGATGAATATGATGAC	TCCCTGAGTGAATAAAGC
Nfkbie	CAATGAGTTGCCAGAGATG	GCCACCAGACCTATAATCC
NfKb1	GTCCACTGTCTGCCTCTC	TCCTTCCTGCCCATAACC
Nfkb2	CACAGAGATGGAGGATTG	GAGGCGAGTAAGAGTTGG
Nik	GCCTCAGCCTCCTCTACC	TGCCAGACTCCTCCTTGC
Crel	GAGACCTGAGACAACTACATG	GCCATTATCTACACAACTGAAG
Rela	CTTGGCAACAGCACAGAC	TCACCAGGCGAGTTATAGC
Rn18s	TCCGATAACGAACGAGAC	CTAAGGGCATCACAGACC
